# Structural basis of death domain signaling in the p75 neurotrophin receptor

**DOI:** 10.7554/eLife.11692

**Published:** 2015-12-08

**Authors:** Zhi Lin, Jason Y Tann, Eddy TH Goh, Claire Kelly, Kim Buay Lim, Jian Fang Gao, Carlos F Ibanez

**Affiliations:** 1 Department of Physiology, National University of Singapore, Singapore, Singapore; 2 Life Sciences Institute, National University of Singapore, Singapore, Singapore; 3 Department of Neuroscience, Karolinska Institute, Stockholm, Sweden; Howard Hughes Medical Institute, Harvard Medical School, United States

**Keywords:** p75, nmr, structure, binding, receptor, Human, Mouse

## Abstract

Death domains (DDs) mediate assembly of oligomeric complexes for activation of downstream signaling pathways through incompletely understood mechanisms. Here we report structures of complexes formed by the DD of p75 neurotrophin receptor (p75^NTR^) with RhoGDI, for activation of the RhoA pathway, with caspase recruitment domain (CARD) of RIP2 kinase, for activation of the NF-kB pathway, and with itself, revealing how DD dimerization controls access of intracellular effectors to the receptor. RIP2 CARD and RhoGDI bind to p75^NTR^ DD at partially overlapping epitopes with over 100-fold difference in affinity, revealing the mechanism by which RIP2 recruitment displaces RhoGDI upon ligand binding. The p75^NTR^ DD forms non-covalent, low-affinity symmetric dimers in solution. The dimer interface overlaps with RIP2 CARD but not RhoGDI binding sites, supporting a model of receptor activation triggered by separation of DDs. These structures reveal how competitive protein-protein interactions orchestrate the hierarchical activation of downstream pathways in non-catalytic receptors.

**DOI:** http://dx.doi.org/10.7554/eLife.11692.001

## eLife digest

Cells have proteins called receptors on their surface that can bind to specific molecules on the outside of the cell. Typically, this binding activates the receptor and the activated receptor then triggers some biochemical changes inside the cell. For many receptors, the portion of the receptor inside the cell is essentially an enzyme that can trigger a biochemical change by itself. Some receptors, however, lack any enzymatic activity, and it is often unclear how these ‘non-catalytic receptors’ trigger changes inside a cell.

A protein called p75 neurotrophin receptor (or p75^NTR^ for short) is a non-catalytic receptor that is expressed when neurons are injured and its activity leads to the death of the neurons and related cells. Inhibiting this non-catalytic receptor is an attractive strategy for limiting the damage caused by diseases of the nervous system. However, the molecular mechanisms behind the activity of p75^NTR^ are not well understood.

Previous biochemical studies set out to answer the question of how p75^NTR^ engages with components of the signaling machinery inside the cell, and found several components that interact with this receptor. Now, Lin et al. have tried to gain a more detailed understanding of those interactions at a molecular level. This involved solving the three-dimensional structures of three protein complexes that involve part of p75^NTR^ (called the “death domain”) and one of two signaling components (called RhoGDI and RIP2).

Two of the protein complexes showed that RIP2 and RhoGDI bind to the receptor’s death domain at partially overlapping sites, although RIP2 binds about 100 times more strongly than RhoGDI.A third protein complex showed an interaction between two copies of the death domain, which involves a surface of the receptor that overlaps with RIP2’s, but not RhoGDI’s, binding site.

These structures, together with the results of other experiments, allowed Lin et al. to propose a model that could explain how p75^NTR^ is activated. First, the two death domains must be separated. Next, RIP2 is recruited to the receptor, and outcompetes and displaces RhoGDI. This change in protein-protein interactions switches the receptor’s signaling from one pathway to the other. Now that these structures are available, they can be used in future experiments to design specific changes in the receptor that would allow researchers to dissect its different activities.

**DOI:** http://dx.doi.org/10.7554/eLife.11692.002

## Introduction

The death domain (DD) is a globular protein module of 80–90 amino acid residues with a characteristic six-helix bundle fold (Feinstein et al., 1995; Ferrao and Wu, 2012). DDs are present in a variety of proteins, including several members of the tumor necrosis factor receptor superfamily (TNFRSF) and a range of intracellular signaling components, such as caspases and kinases. The DD family includes four subfamilies of structurally related domains, including the canonical DD, the death effector domain (DED), the caspase recruitment domain (CARD), and the pyrin domain (PYD). DD-containing proteins play central roles in apoptotic and inflammatory signaling through the formation of oligomeric protein complexes, and several disease-causing mutations have been mapped to DD interfaces (Park et al., 2007). All DD complexes described so far involve homotypic interactions between DDs of the same subfamily (e.g., DD with DD, CARD with CARD, etc.). All known DD interactions belong to one of three types (I to III), each mediated by conserved asymmetric interfaces in the interacting DDs (Park, 2011; Park et al., 2007; Weber and Vincenz, 2001). Heterotypic complexes between DDs from different subfamilies have not yet been described and, aside from a few structures of DDs bound to small polypeptides, no complexes of DDs with proteins outside the DD superfamily have been reported. Thus, type I, II, and III interactions between DDs are thought to represent the predominant mechanism of oligomerization and complex formation for DD-containing proteins.

The cytoplasmic domain of the p75 neurotrophin receptor (p75^NTR^, also known as NGFR and TNFRSF16) contains a C-terminal DD connected to the transmembrane (TM) domain by a 60-residue-long linker region (Liepinsh, 1997). p75^NTR^ is a receptor for members of the neurotrophin family, such as nerve growth factor (NGF) and brain-derived neurotrophic factor (BDNF) (Dechant and Barde, 2002; Roux and Barker, 2002). In addition to the neurotrophins, a number of other extracellular ligands can also engage or signal through p75^NTR^, including the beta-amyloid peptide (Knowles et al., 2009; Perini et al., 2002), the rabies virus glycoprotein (Tuffereau et al., 1998), and various myelin-derived polypeptides (Wang et al., 2002; Wong et al., 2002). p75^NTR^ may function alone or in conjunction with other transmembrane proteins to allow ligand binding and intracellular signaling. These proteins include members of the Trk family of receptor tyrosine kinases, members of the Vps10p family of sorting receptors, such as Sortilin, and the Nogo receptor, which promotes binding to myelin-derived ligands (Underwood and Coulson, 2008). p75^NTR^ can engage different intracellular pathways, of which the best characterized are the RhoA pathway, which regulates axon growth, collapse and degeneration (Park et al., 2010; Yamashita et al., 1999; Yamashita and Tohyama, 2003), the NF-kB pathway, which contributes to cell survival (Carter et al., 1996; Khursigara et al., 2001; Vicario et al., 2015), and the c-Jun kinase (JNK) or caspase pathway, which mediates apoptotic cell death (Friedman, 2000; Yoon et al., 1998). p75^NTR^ signaling through any of those three pathways requires a functional DD (Charalampopoulos et al., 2012). Expression of p75^NTR^ increases in a number of neurodegenerative diseases and upon injury or stress conditions, where it contributes to neuronal and glial cell damage, axonal degeneration, and synaptic dysfunction (Ibanez and Simi, 2012). Inhibition of p75^NTR^ signaling has emerged as an attractive strategy for limiting neural damage in neurodegeneration and nerve injury.

The mechanism of activation of p75^NTR^ in response to neurotrophins involves a conformational rearrangement of disulfide-linked receptor dimers, resulting in the separation of intracellular DDs (Vilar et al., 2009). Fluorescence resonance energy transfer (FRET) experiments have shown that the two DDs in the p75^NTR^ dimer are in close proximity to each other (high FRET state) and that NGF binding induces a decrease in FRET signal (Vilar et al., 2009). Disruption of this conformational change through mutation of a conserved cysteine residue in the TM domain prevents p75^NTR^ signaling in response to neurotrophins (Vilar et al., 2009). p75^NTR^ lacks an associated catalytic activity. Similar to other members of the TNFRSF, signaling by p75^NTR^ proceeds via ligand-induced recruitment and release of cytoplasmic effectors to and from its intracellular domain. Ligand-induced separation of p75^NTR^ DDs may allow the recruitment of intracellular components for downstream signal propagation. Although a variety of intracellular proteins have been reported to interact with p75^NTR^, the molecular mechanisms by which the receptor engages different signaling pathways remain unclear. To begin addressing these questions, our laboratory performed a comprehensive structure–function study of the p75^NTR^ DD that resulted in the identification of three sets of solvent-exposed residues that are critical for p75^NTR^’s ability to engage the RhoA, NF-kB and JNK/cell death pathways, respectively (Charalampopoulos et al., 2012). Receptor mutants that are selectively deficient in one pathway but not others were generated, demonstrating that different signaling outputs can be genetically separated in p75^NTR^. Understanding how such interfaces relate to each other and to the mechanism of receptor activation has remained an important challenge.

In this study, we have undertaken a structural biology approach to elucidate the molecular mechanisms underlying downstream signaling mediated by the DD in p75^NTR^. We have determined the solution structures of the p75^NTR^ DD in complex with RhoGDI (Rho guanine nucleotide dissociation inhibitor), which links the receptor to the RhoA pathway (Yamashita et al., 1999; Yamashita and Tohyama, 2003), or with the CARD domain of RIP2 kinase, which is necessary for p75^NTR^ coupling to the NF-kB pathway (Charalampopoulos et al., 2012; Khursigara et al., 2001). We have also solved the solution structure of the p75^NTR^ DD homodimer, revealing the DD surface that is occluded prior to neurotrophin-mediated receptor activation. These structures uncover novel heterotypic DD interactions, not previously seen in other DD-containing complexes, and reveal the molecular mechanisms underlying the early stages of p75^NTR^ activation and downstream signaling.

## Results

### Solution structure of the complex between the p75^NTR^ DD and RhoGDI

**Figure 1.**
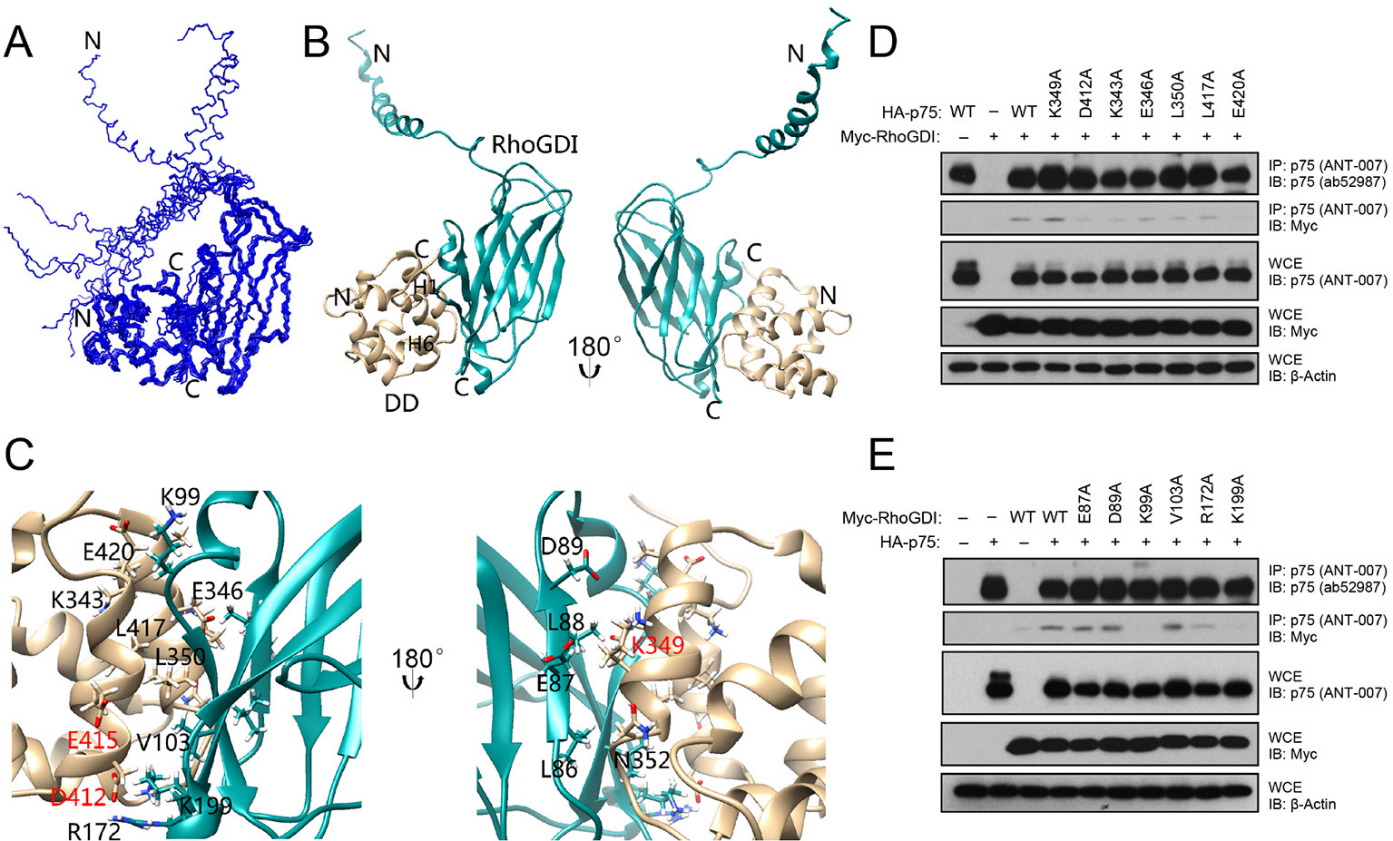
Solution structure of the complex between the p75^NTR^ DD and RhoGDI. (**A**) Superposition of backbone heavy atoms of the 10 lowest-energy structures of the human p75^NTR^ DD:RhoGDI complex. N- and C-termini are indicated. (**B**) Ribbon drawing of the lowest-energy conformer. Light brown, p75^NTR^ DD; Cyan, RhoGDI. N- and C-termini, as well as DD helices H1 and H6 are indicated. (**C**) Details of binding interface in the complex viewed in the same orientations as panel B, respectively. Key residues at the binding interface are labeled and depicted as stick models. Red labels denote interface residues functionally validated in our earlier mutagenesis study (Charalampopoulos et al., 2012). (**D**) Co-immunoprecipitation of wild type (**WT**) and DD point mutants of human p75^NTR^ with Myc-tagged RhoGDI in transfected HEK 293 cells. Antibodies used for immunoprecipitation (**IP**) and Western blotting (**WB**) are indicated. WCE, whole cell lysate. The immunoblots shown are representative of three independent experiments. (**E**) Co-immunoprecipitation of WT and point mutants of Myc-tagged human RhoGDI with p75^NTR^ in transfected HEK 293 cells. Antibodies used for immunoprecipitation (**IP**) and Western blotting (**WB**) are indicated. WCE, whole cell extract. The immunoblots shown are representative of three independent experiments. **DOI:** http://dx.doi.org/10.7554/eLife.11692.004

RhoGDI interacts constitutively with the DD of unliganded p75^NTR^, linking the receptor to the RhoA pathway (Yamashita and Tohyama, 2003). Neurotrophin binding induces the release of RhoGDI from p75^NTR^ and decreases RhoA activity (Gehler et al., 2004; Yamashita et al., 1999; Yamashita and Tohyama, 2003). Using biochemical and cell-based assays, we have previously identified solvent-exposed residues in the p75^NTR^ DD that are critical for its interaction with RhoGDI and RhoA activation, including residues in helices H1 and H6 (Charalampopoulos et al., 2012). In order to obtain a molecular understanding of this interaction, we determined the solution structure of the human p75^NTR^ DD:RhoGDI complex by multidimensional nuclear magnetic resonance (NMR) spectroscopy (Figure 1—figure supplement 1 and Table 1). We note that, unless otherwise indicated, all amino acid residue numbering in this study corresponds to the human forms of the respective proteins. Human and rat p75^NTR^ DD share more than 90% sequence identity—including all functionally relevant residues—and an essentially identical three-dimensional structure with an overall RMSD of 1.7 Å ((Liepinsh, 1997) and this study). The ensemble of the 10 lowest-energy conformers of the DD:RhoGDI complex and a representative structure are depicted in Figure 1A,B. p75^NTR^ DD in the complex consists of one 3_10_-helix followed by six α-helices and its global fold is very similar to that of our previously described structure of rat p75^NTR^ DD (Liepinsh, 1997). In the complex, the C-terminal domain of RhoGDI primarily displays an immunoglobulin-like fold similar to previously described structures of this protein (Longenecker et al., 1999). Residues Glu^40^ to Gly^57^ in the RhoGDI N-terminal domain fold into a long helix, which is not involved in p75^NTR^ DD binding and remains flexible in the complex (Figure 1A). Removal of this N-terminal domain did not affect RhoGDI binding to p75^NTR^ DD (Figure 1—figure supplement 2). Inspection of the interface in the complex showed that it is mainly formed by α-helices H1 and H6 of the p75^NTR^ DD and β-strands S2, S3, S9 and α-helix H2 of RhoGDI, forming a small hydrophobic core surrounded by polar residues (Figure 1C). Charged residues play an important role in the binding interface and high concentration of salt (i.e., greater than 200 mM NaCl) can almost completely disrupt p75^NTR^ DD:RhoGDI interaction in vitro (Figure 1—figure supplement 1C). It is gratifying to note that all functional DD determinants that we have previously identified by site-directed mutagenesis clustered at the DD:RhoGDI interface of the complex structure (labeled red in Figure 1C,D). The structure of the DD:RhoGDI complex offered an opportunity to address the functional importance of a larger set of residues in the p75^NTR^ DD as well as in RhoGDI. Co-immunoprecipitation experiments were performed in cells transfected with constructs of full-length p75^NTR^ and RhoGDI carrying different point mutations in selected residues. Alanine substitution of individual amino acid residues likely uncovers only those side chains making the most critical contribution to the binding interface. In the p75^NTR^ DD, substitution of either Asp^412^, Lys^343^ or Glu^420^ was found to significantly diminish interaction with RhoGDI (Figure 1D). In RhoGDI, substitution of Lys^99^ or Lys^199^ abolished its interaction with p75^NTR^ (Figure 1E). In agreement with this, the structure of the complex shows that these two positively charged side chains make charge interactions with Glu^420^ and Asp^412^, respectively, in the p75^NTR^ DD (Figure 1C).

**Table 1.** NMR and refinement statistics for p75^NTR^ DD complexes and RIP2 CARD. **DOI:** http://dx.doi.org/10.7554/eLife.11692.003

NMR distance and dihedral constraints	DD:RhoGDI	RIP2 CARD	DD:CARD	DD:DD
Distance constraints				
Total NOE	3525	2107	3760	3344
Intra-residue	809	436	798	728
Inter-residue				
Sequential ( *i* – *j* = 1)	1054	656	1130	986
Medium-range ( *i* – *j* ≤ 4)	665	589	1016	892
Long-range ( *i* – *j* ≥ 5)	945	426	771	706
Intermolecular NOE	52	-	45	32
Total dihedral angle restraints ^a^	222	132	260	280
				
Structure Statistics				
Violations (mean and s.d.)				
Distance constraints (Å)	0.36 ± 0.02	0.25 ± 0.01	0.36 ± 0.03	0.28 ± 0.01
Dihedral angle constraints (º)	3.50 ± 0.46	2.75 ± 0.28	3.37 ± 0.28	2.86 ± 0.59
Max. dihedral angle violation (º)	4.16	3.25	3.77	4.28
Max. distance constraint violation (Å)	0.39	0.26	0.44	0.29
Ramachandran Plot (allowed region)	99.8%	99.5%	99.8%	99.9%
Average RMSD (Å) ^b^				
Heavy atoms	0.91 ± 0.12	0.83 ± 0.06	0.99 ± 0.08	0.77 ± 0.06
Backbone atoms	0.55 ± 0.13	0.36 ± 0.05	0.66 ± 0.09	0.43 ± 0.02

^a^ Dihedral angle constraints were generated by TALOS based on C_α_ and C_β_ chemical shifts.

^b^ Average r.m.s. deviation (RMSD) to the mean structure was calculated among 10 refined structures. Superimposing residues for DD:RhoGDI, RIP2 CARD, DD:CARD, and DD:DD are 334–421 of DD with 70–204 of RhoGDI, 436–523 of RIP2 CARD, 334–420 of DD with 435–534 of RIP2 CARD and 334–421 of DD respectively. The total AMBER energy for DD:RhoGDI, RIP2 CARD, DD:CARD, and DD:DD are –9884 ± 41, –4437 ± 32, –7706 ± 32 and –7161 ± 18 kcal/mol respectively.

**Figure 1—figure supplement 1.**
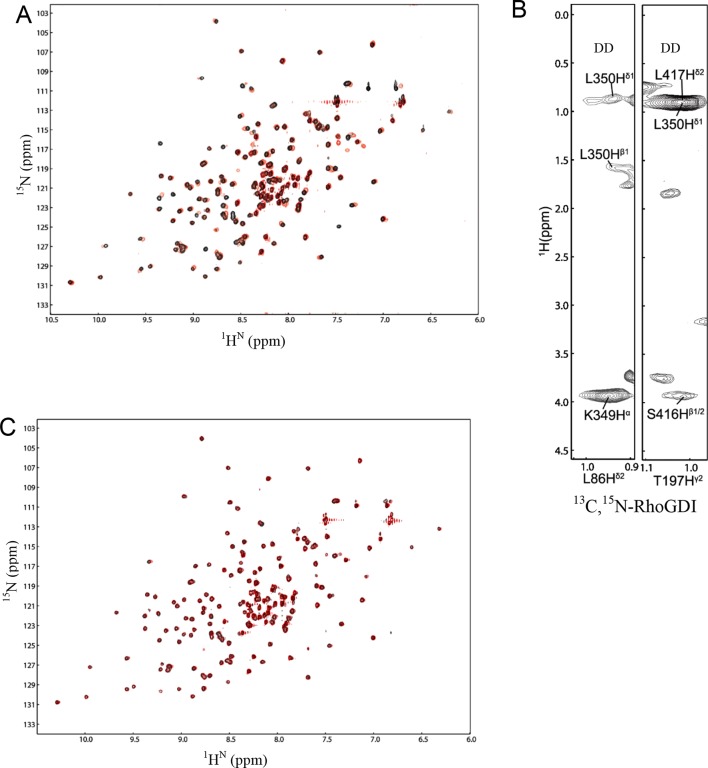
NMR spectra of DD:RhoGDI complex in 20 mM HEPES at 28°C and pH 6.9. (**A**) [^1^H-^15^N] HSQC spectra of ^15^N-RhoGDI in the absence (black) and presence (red) of p75^NTR^ DD. The concentration of RhoGDI and p75^NTR^ DD was 0.5 and 2 mM, respectively. (**B**) Representative slices from the ^13^C,^15^N-filtered 3D NOESY spectrum. (**C**) [^1^H-^15^N] HSQC spectra of ^15^N-RhoGDI in complex with p75^NTR^ DD in the absence (black) and presence (red) of 250 mM NaCl. **DOI:** http://dx.doi.org/10.7554/eLife.11692.005

**Figure 1—figure supplement 2.**
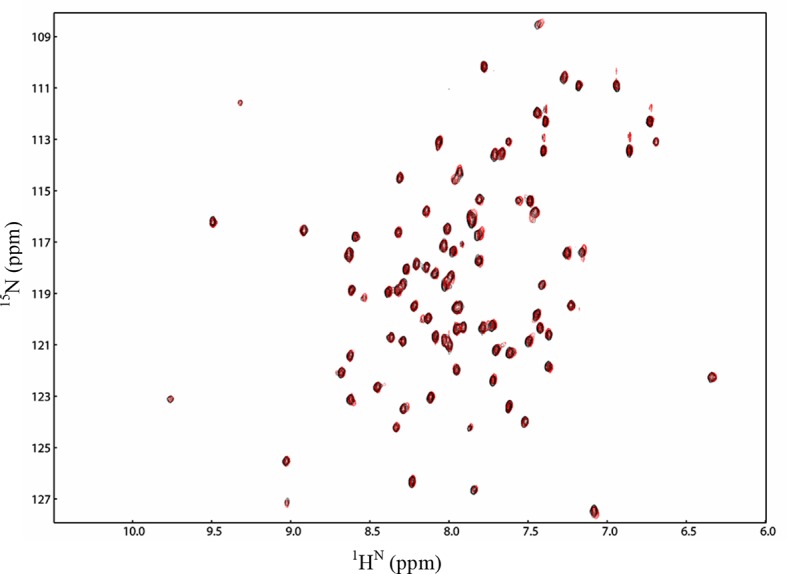
The N-terminal domain of RhoGDI does not bind to p75^NTR^ DD. [^1^H-^15^N] HSQC spectra of p75^NTR^ DD in the presence of RhoGDI (black) and RhoGDI without N-terminal domain (RhoGDI ΔN, red) at 28°C and pH 6.9. Molar ratio of DD to RhoGDI or to RhoGDI ΔN was 1:4. **DOI:** http://dx.doi.org/10.7554/eLife.11692.006

**Figure 2.**
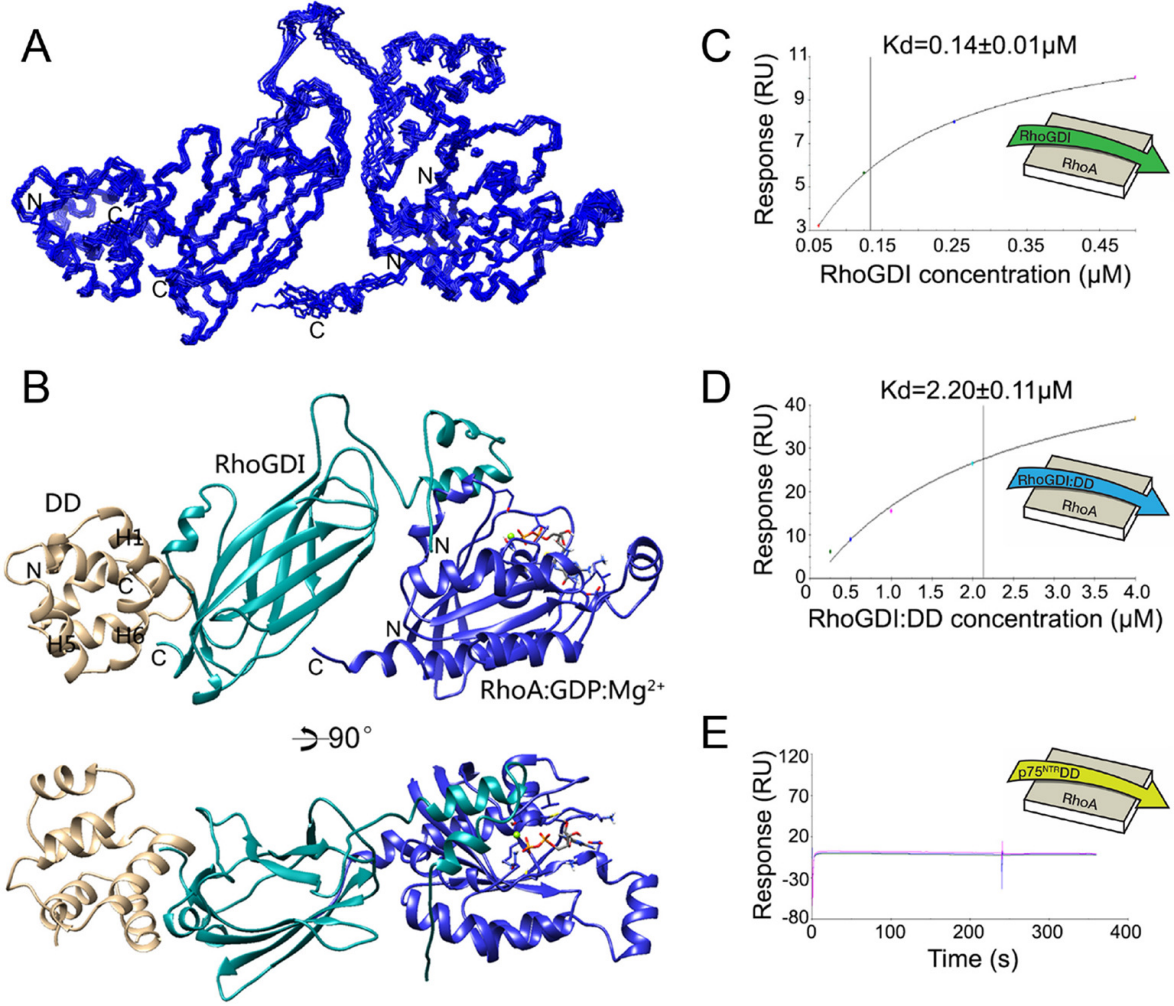
Structural model of tripartite complex between p75^NTR^ death domain, RhoGDI and RhoA. (**A**) Superposition of backbone traces of the 10 lowest-energy structures of p75^NTR^ DD:RhoGDI:RhoA tripartite complex. N- and C-termini are indicated. (**B**) Ribbon diagram of a representative structure of p75^NTR^ DD:RhoGDI:RhoA heterotrimer complex. Light brown, p75^NTR^ DD; Cyan, RhoGDI; Blue, RhoA. Mg^2+^ and GDP appear in ball-and-stick models. p75^NTR^ DD helices H1, H5 and H6 as well as N- and C-termini are indicated. (**C**) Binding of RhoGDI to immobilized RhoA:GDP:Mg^2+^ measured by surface plasmon resonance (**SPR**). Binding affinity was determined by steady-state analysis. One binding site model was used for fitting of SPR data. The sensorgram shown is representative from three independent experiments. (**D**) Binding of RhoGDI complexed with p75^NTR^ DD (molar ratio 1:2) to immobilized RhoA:GDP:Mg^2+^ measured by SPR. Binding affinity was determined by steady-state analysis. One binding site model was used for fitting of SPR data. The sensorgram shown is representative from three independent experiments. (**E**) Sensorgram showing lack of interaction between p75^NTR^ DD (tested at 125–500 nM) and immobilized RhoA:GDP:Mg^2+^. The sensorgram shown is representative from three independent experiments. **DOI:** http://dx.doi.org/10.7554/eLife.11692.007

Analysis of the p75^NTR^ DD:RhoGDI complex and a previously described crystal structure of the RhoGDI:RhoA(GDP) complex (Longenecker et al., 1999) indicated that p75^NTR^ DD and RhoA interact with different surfaces in RhoGDI, located at opposite sides of the molecule. The two distant binding sites on RhoGDI suggested that a heterotrimer complex p75^NTR^ DD:RhoGDI:RhoA may be structurally feasible. We investigated this by performing HADDOCK calculations (Dominguez et al., 2003) using our solution structure of p75^NTR^ DD from its complex with RhoGDI and the crystal structure of the RhoGDI:RhoA(GDP) complex. Multiple refinements converged to a mean backbone root mean square deviation (RMSD) of 0.64 ± 0.05 Å (Figure 2A,B and Table 2). Ramachandran plot analysis of the docking model indicated that the trimer structure, including the two intermolecular interfaces, still occupies the energetically preferred conformation. In the tripartite complex, the N-terminal domain of RhoGDI folded into two helices and bound to RhoA(GDP), similar to its conformation in the RhoGDI:RhoA(GDP) complex (Longenecker et al., 1999). The DD binding site on RhoGDI remained nearly identical to that in the DD:RhoGDI complex. This analysis shows that interaction of the three proteins can indeed occur simultaneously and explains previous biochemical studies showing that RhoA can be co-immunoprecipitated with p75^NTR^ in the presence of RhoGDI (Yamashita et al., 1999). Using the purified proteins, we determined the binding affinity of the RhoGDI:RhoA complex by surface plasmon resonance (SPR). Titration of RhoGDI onto immobilized RhoA, yielded a binding *K*_d_ of 0.14 ± 0.01 μM, which is in agreement with previous measurements (Tnimov et al., 2012) (Figure 2C). Interestingly, when RhoGDI was precomplexed with purified p75^NTR^ DD, the *K*_d_ was 2.2 ± 0.11 μM (Figure 2D), indicating that binding to the p75^NTR^ DD decreases the affinity of the RhoGDI:RhoA interaction by about 15-fold. We note that, in the absence of RhoGDI, no binding between p75^NTR^ DD and RhoA could be detected in these experiments (Figure 2E). These results suggest that RhoGDI binding to the p75^NTR^ DD weakens its interaction with RhoA, a step which may facilitate RhoA activation.

**Figure 2—figure supplement 1.**
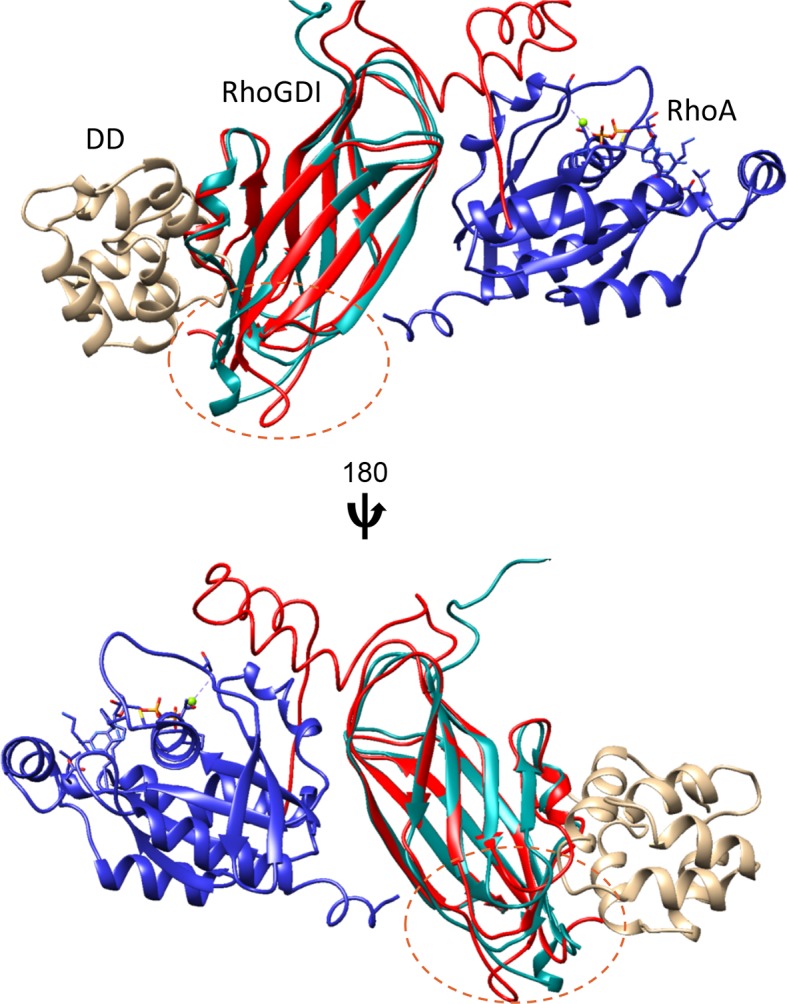
Local structural differences in RhoGDI after interaction with either p75^NTR^ DD or RhoA:GDP. Ribbon diagram of RhoGDI from the complex with p75^NTR^ DD (light brown) is shown in cyan, and from the complex with RhoA (blue) in red (from Longenecker et al., 1999). Main structural differences in RhoGDI are indicated by the dotted line circle. **DOI:** http://dx.doi.org/10.7554/eLife.11692.008

**Table 2.** Structural statistics for the 10 lowest-energy structures of p75^NTR^DD:RhoGDI:RhoA Trimer and Hexamer^a^. **DOI:** http://dx.doi.org/10.7554/eLife.11692.009

	Trimer	Hexamer
Backbone RMSD (Å)			
From the mean, full complex	0.61 ± 0.25	0.59 ± 0.20	
From the mean, all interfaces	0.58 ± 0.22	0.48 ± 0.13	
Total energy (kcal/mol)	-20721 ± 137	-37682 ± 210	
Ramachandran plot (%)^b^			
Residues in the most favored regions	82.2	84.7	
Residues in additional allowed regions	14.1	12.5	
Residues in generously allowed regions	1.9	1.9	
Residues in disallowed regions	1.8	0.9	

^a^Structural statistics for the 10 lowest-energy conformers were obtained from HADDOCK calculation using NOEs between DD and RhoGDI.

^b^Ramachandran analysis was carried out using PROCHECK-NMR.

### Solution structure of the complex between the p75^NTR^ DD and RIP2 CARD

**Figure 3.**
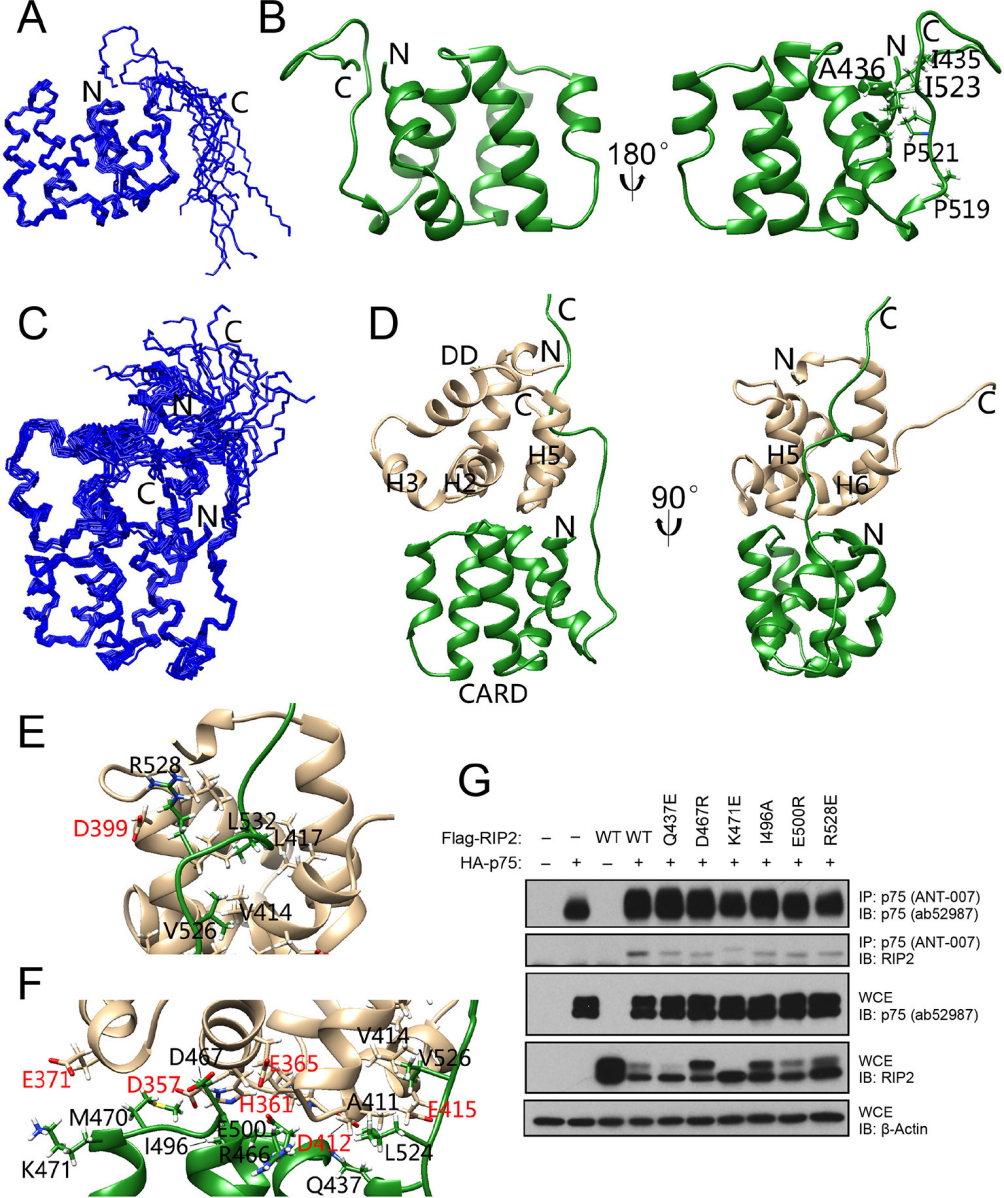
Solution structure of RIP2 CARD and its complex with p75^NTR^ DD. (**A**) Superposition of backbone heavy atoms of the 10 lowest-energy structures of human RIP2 CARD. N- and C-termini are indicated. (**B**) Ribbon drawing of the lowest-energy conformer of human RIP2 CARD. N- and C-termini, as well as selected residues in the C-terminal tail are indicated. (**C**) Superposition of backbone heavy atoms of the 10 lowest-energy structures of the human p75^NTR^ DD:RIP2 CARD complex. N- and C-termini are indicated. (**D**) Ribbon drawing of the lowest-energy p75^NTR^ DD:RIP2 CARD conformer. Light brown, p75^NTR^ DD; Green, RIP2 CARD. N- and C-termini, as well as DD helices H2, H3, and H5 are indicated. (**E** and **F**) Details of binding interface in the complex viewed in the same orientations as panel D, respectively. Key residues at the binding interface are labeled and depicted as stick models. Red labels denote interface residues functionally validated in our earlier mutagenesis study (Charalampopoulos et al., 2012). (**G**) Co-immunoprecipitation of wild type (**WT**) and point mutants of Flag-tagged human RIP2 with p75^NTR^ in transfected HEK 293 cells. In the overexpression conditions used for this experiment, interaction of RIP2 with p75^NTR^ was constitutive in the absence of ligand. Antibodies used for immunoprecipitation (**IP**) and Western blotting (**WB**) are indicated. WCE, whole cell extract. The immunoblots shown are representative of three independent experiments. **DOI:** http://dx.doi.org/10.7554/eLife.11692.010

NGF binding to p75^NTR^ elicits the recruitment of RIP2 kinase to the receptor. Recruitment of RIP2 is required for regulation of the NF-kB pathway by p75^NTR^. Previous biochemical studies established that the interaction between p75^NTR^ and RIP2 is mediated by their DD and CARD domains, respectively (Khursigara et al., 2001). The RIP2 CARD consists of 107 amino acids and is located in the C-terminal of the protein. It connects to the N-terminal kinase domain via a linker of 120 amino acids. We determined the NMR structure of human RIP2 CARD in monomeric form (Figure 3A,B and Table 1). The solution structure of RIP2 CARD comprises an arrangement of six α-helices followed by one short 3_10_-helix, all tightly packed around a hydrophobic core. A C-terminal tail of 17 amino acids (Leu^524^-Met^540^) follows the CARD and is unstructured and flexible in solution. A unique segment (Gln^518^-Ile^523^) between the C-terminal tail and the 3_10_-helix contains two structural disruptor residues (i.e., Pro^519^ and Pro^520^, Figure 3B) and lacks a secondary structure, but its orientation was well-defined in the NMR structure. A number of hydrophobic residues (e.g., Ile^523^, Figure 3B) in this segment closely interact with the first and the last α-helices in the RIP2 CARD. Structural comparison using the DALI server (Holm and Rosenström, 2010) showed that the most similar structure to RIP2 CARD was the CARD of nucleotide-binding oligomerization domain-containing protein 1 (NOD1), with a *Z*-score between 9 and 11. NOD1 and RIP2 have been shown to interact with each other through their CARDs to propagate immune signaling (Mayle et al., 2014). The two CARDs share similar structural features, including a similar arrangement of all but the last of the α-helices, which displays different local secondary structures in the two proteins (Figure 3—figure supplement 1A). Despite their folding similarities, the two CARDs exhibit significantly different surface characteristics. Particularly, RIP2 CARD has many more charged residues on its surface than its NOD1 counterpart (Figure 3—figure supplement 1B,C). Different electrostatic surfaces will confer different interaction specificities, a common feature among members of the DD superfamily, including the CARD subfamily.

**Figure 3—figure supplement 1.**
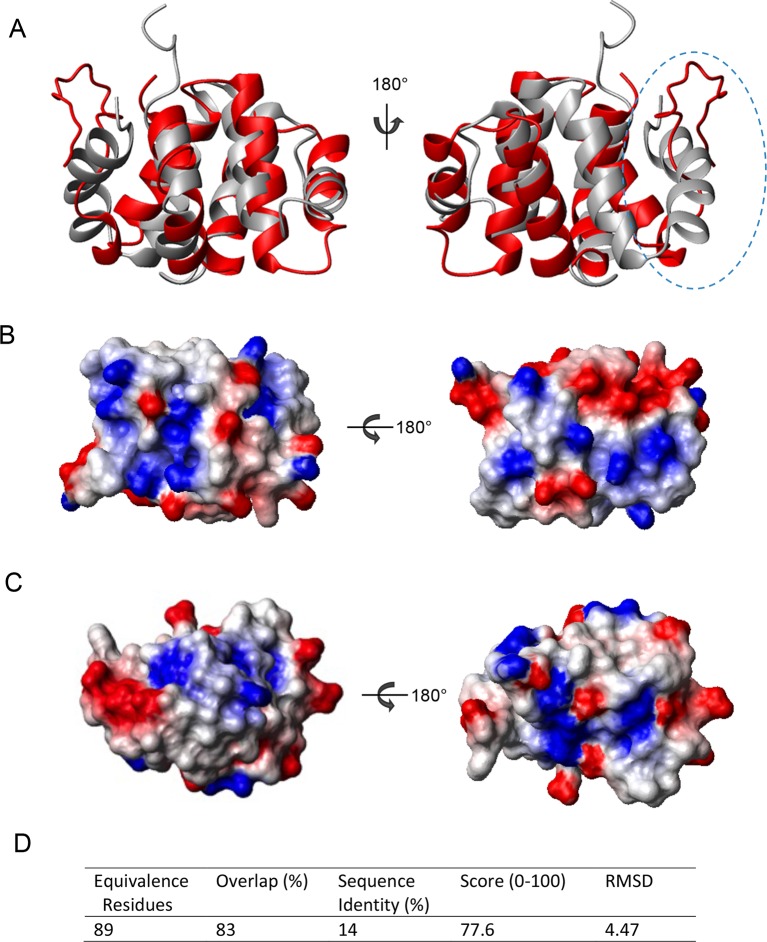
Structure comparison of CARD domains using a sequential structure alignment program (http://v3-4.cathdb.info/). (**A**) Overlap of RIP2 CARD (red) and NOD1 CARD (gray, PDB ID: 2B1W). (**B**) Surface charge of RIP2 CARD without C-terminal tail. Positive charge surface is colored in blue, negative in red and noncharged in white. (**C**) Surface charge of NOD1 CARD. (**D**) Statistics of pairwise alignment of CARDs from RIP2 and NOD1. **DOI:** http://dx.doi.org/10.7554/eLife.11692.011

**Figure 3—figure supplement 2.**
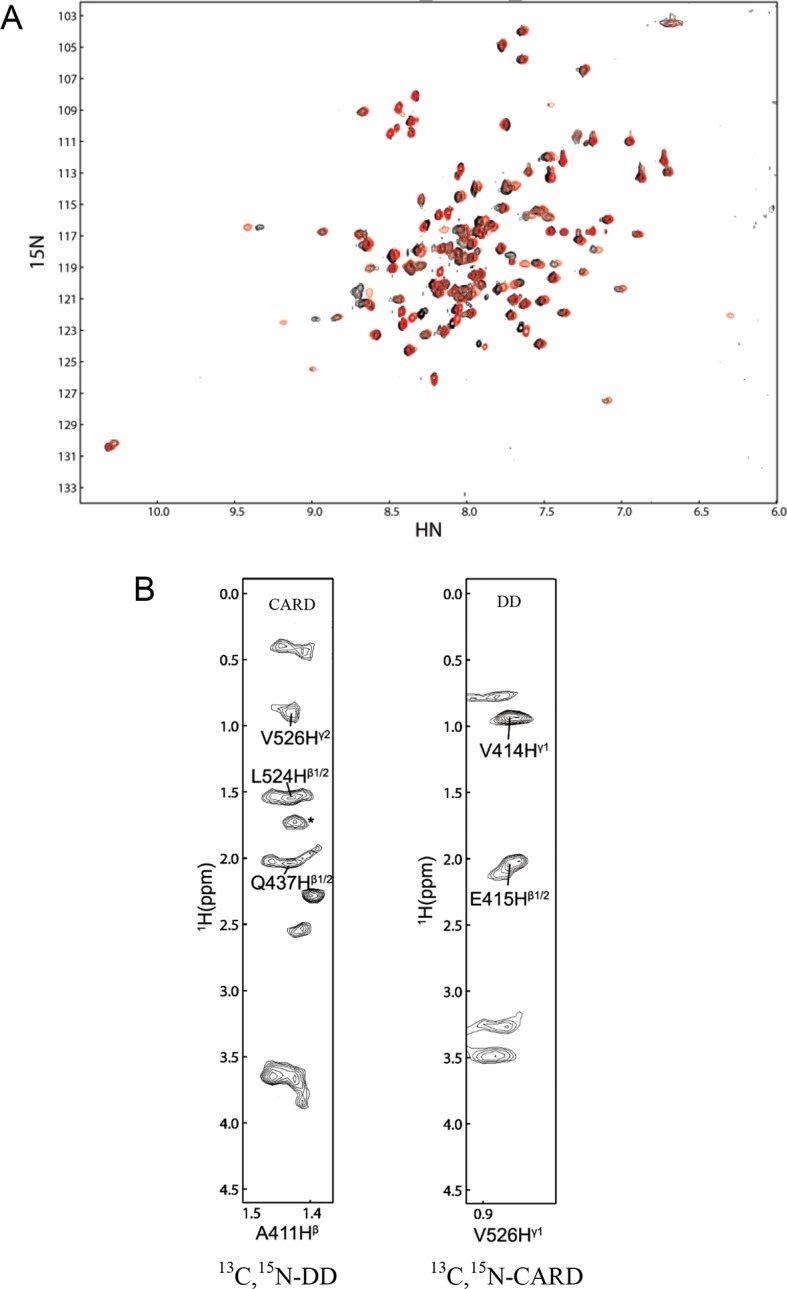
NMR Spectra of DD:CARD complex. (**A**) [^1^H-^15^N] HSQC spectra of p75^NTR^ DD in water in the absence (black) and presence (red) of RIP2 CARD. (**B**) Representative slices from the ^13^C,^15^N-filtered 3D NOESY spectra. Asterisk denotes ambiguous NOE peak. **DOI:** http://dx.doi.org/10.7554/eLife.11692.012

**Figure 3—figure supplement 3.**
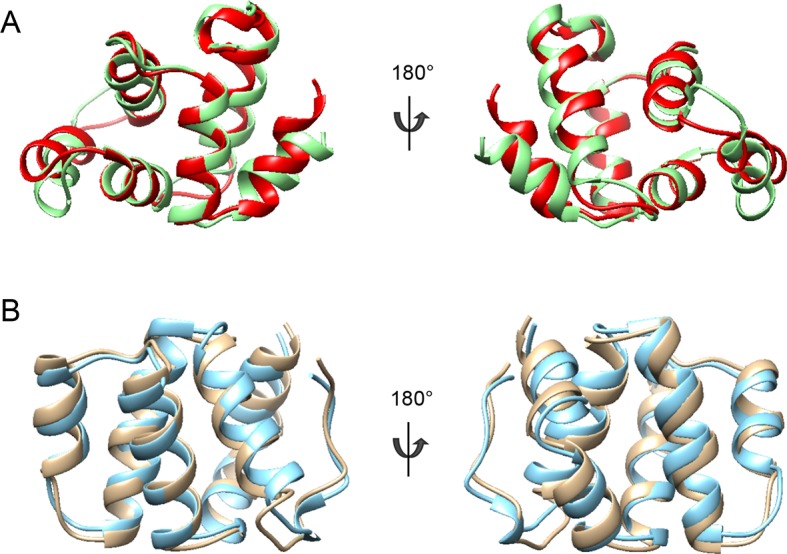
Structural comparisons of p75^NTR^ DD and RIP2 CARD domains. (**A**) Structural comparison between p75^NTR^ DD from the DD:RhoGDI (red) and DD:CARD (green) complexes. (**B**) Structural comparison between monomeric RIP2 CARD (cyan) and RIP2 CARD from the DD:CARD complex (light brown). **DOI:** http://dx.doi.org/10.7554/eLife.11692.013

**Figure 3—figure supplement 4.**
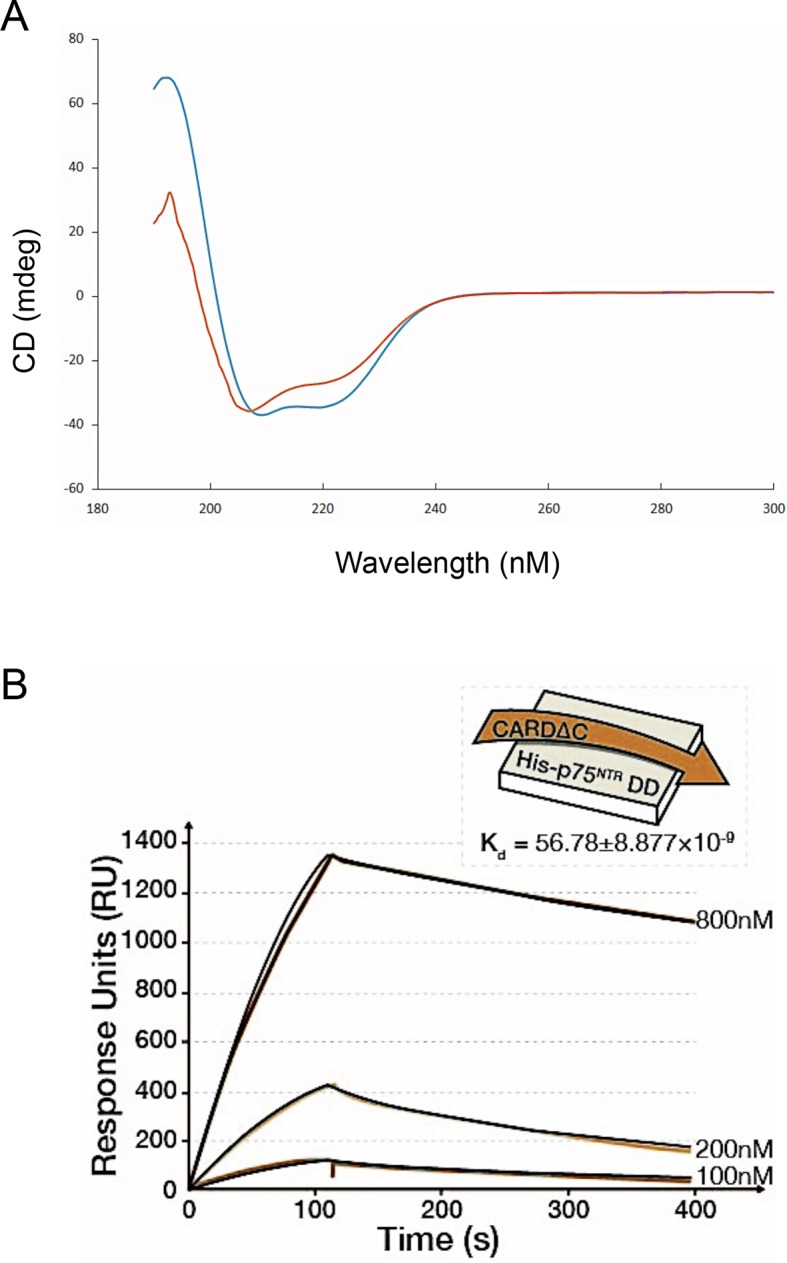
The C-terminal tail of RIP2 CARD contributes to its interaction with the p75^NTR^DD. (**A**) CD spectra of CARD (orange) and CARD ΔC mutant lacking the C-terminal tail (blue). (**B**) Sensorgram of binding kinetics of CARD ΔC binding to p75^NTR^ DD at pH 7.0. One binding site model was used for fitting of SPR data. **DOI:** http://dx.doi.org/10.7554/eLife.11692.014

In order to obtain a molecular understanding of the p75^NTR^ DD:RIP2 CARD interaction, we determined the NMR structure of the complex. Figure 3C,D present the three-dimensional solution structure of this complex obtained from the NMR experimental restraints (Table 1 and Figure 3—figure supplement 2). The core helical structure of the p75^NTR^ DD in the p75^NTR^ DD:RIP2 CARD complex was similar to that in the p75^NTR^ DD:RhoGDI complex, with a pairwise RMSD of ~1.9 Å (Figure 3—figure supplement 3A). The orientation of α-helices H3 and H6 changed by approximately 7°–8°. Similarly, pairwise RMSD between RIP2 CARD in monomeric form and in complex with the p75^NTR^ DD was ~1.5 Å (Figure 3—figure supplement 3B). The main interface in the core structure of the p75^NTR^ DD:RIP2 CARD complex involved α-helices H2, H3, H5, H6, and H5–H6 loop of p75^NTR^ DD and α-helices H1, H3–H4, and H5–H6 loops of RIP2 CARD (Figure 3E,F). Both electrostatic and hydrophobic interactions contribute to the p75^NTR^ DD:RIP2 CARD interface. Interestingly, the C-terminal tail of RIP2 CARD was better defined in its complex with the p75^NTR^ DD compared to its monomeric form, although the last six amino acids still remained flexible. The C-terminal tail bound to α-helices H1, H5, and H6 of p75^NTR^ DD, through both hydrophobic and charged interactions (Figure 3D and Figure 3—figure supplement 3B). In our previous site-directed mutagenesis studies of the p75^NTR^ DD, we had identified residues in helices H2 (Asp^357^, His^361^, and Glu^365^), H3 (Gln^369^ and Glu^371^), H5 (Asp^399^), and H6 (Asp^412^ and Glu^415^) as being critical for its interaction with RIP2 (Charalampopoulos et al., 2012). We were pleased to note that all these residues mapped to the DD:CARD binding interface defined in our NMR structure of the complex (labeled red in Figure 3E,F). The NMR structure of the DD:CARD complex offered an opportunity to address the functional importance of residues in the RIP2 CARD. Co-immunoprecipitation experiments were performed in cells transfected with constructs of full-length p75^NTR^ and RIP2, the latter carrying different point mutations in selected residues of the CARD. We found that individual substitution of interface residues Gln^437^, Asp^467^, Lys^471^, Ile^496^, Glu^500^, or Arg^528^, significantly diminished RIP2 interaction with p75^NTR^ (Figure 3G).

### Differential binding of RhoGDI and RIP2 CARD to the p75^NTR^ DD

**Figure 4.**
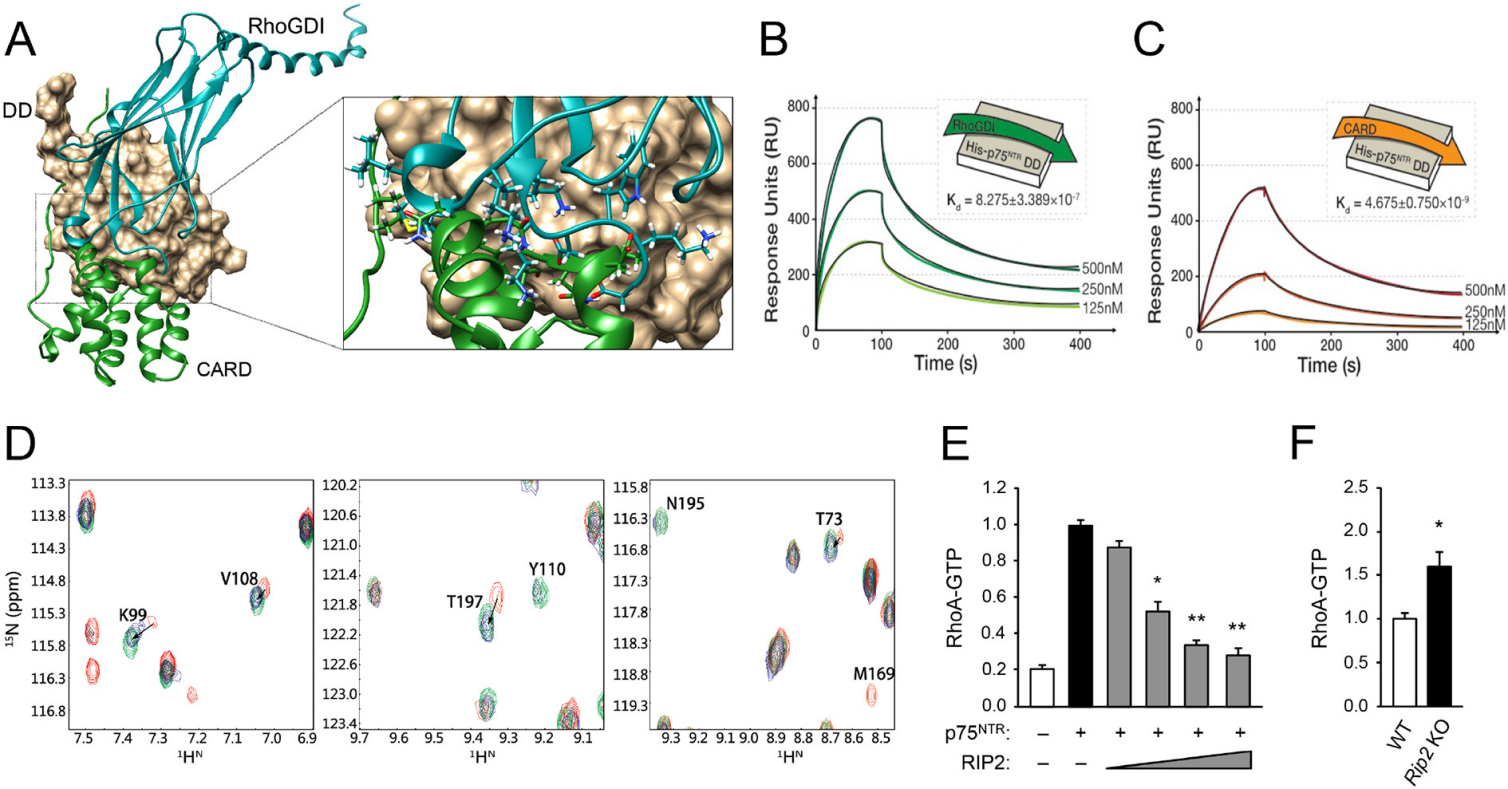
Structural basis for competitive interaction between RIP2 and RhoGDI on the p75^NTR^ DD. (**A**) Surface representation of p75^NTR^ DD (light brown) with overlapped ribbon drawings of RhoGDI (cyan) and RIP2 CARD (green). The expanded view shows detail of the overlapping interfaces demonstrating steric clashes between residues in RhoGDI and CARD (highlighted as stick models). (**B** and **C**) Binding of RhoGDI (B) and RIP2 CARD (including C-terminal tail) (C) to captured His-tagged p75^NTR^ DD measured by SPR. Colored lines represent experimentally recorded values at different concentrations and black lines are fitting data. Binding affinities were determined by kinetic analysis using one binding site model was used for fitting of SPR data. The sensorgrams shown are representative from three independent experiments. (**D**) [^1^H-^15^N] HSQC spectra of ^15^N-RhoGDI showing the ability of RIP2 to displace RhoGDI from the p75^NTR^ DD. The panels show details of different regions of the spectra for RhoGDI alone (green), RhoGDI in the presence of p75^NTR^ DD (red), and RhoGDI in the presence of both p75^NTR^ DD and RIP2 CARD (blue). Representative RhoGDI residues located in and/or close to the DD:RhoGDI interface are labeled. Arrows denote shifts in the spectra of labeled RhoGDI residues upon addition of p75^NTR^ DD and RIP2 CARD. All the spectra were recorded at pH 6.9 and 28°C. The concentrations of RhoGDI, p75^NTR^ DD and RIP2 CARD were 0.05, 0.2, and 0.2 mM respectively. (**E**) Analysis of RhoA-GTP levels in lysates of HEK293 cells transfected with p75^NTR^ and RIP2 expression constructs, as indicated. Increasing concentrations of RIP2 construct is indicated. The histogram shows average RhoA-GTP levels (from triplicate measurements) normalized to p75^NTR^ alone. Protein expression levels were controlled by Western blotting (not shown). * p<0.01 vs. p75^NTR^ alone (*t*-test). (**F**) Analysis of RhoA-GTP levels in cerebellar extracts prepared from P7 *Rip2* knockout mice and wild type littermates (**WT**). The histogram shows average RhoA-GTP levels in WT (*N *= 3) and KO (*N *= 4) animals normalized to WT levels. *p<0.05 vs. WT (*t*-test). **DOI:** http://dx.doi.org/10.7554/eLife.11692.015

Comparison of the DD interfaces used for binding to RhoGDI and RIP2 CARD showed that these shared partially overlapping binding sites on p75^NTR^ DD (Figure 4A), indicating that RIP2 and RhoGDI cannot bind to the p75^NTR^ DD simultaneously due to steric hindrance. This is in agreement with our previous biochemical studies that identified overlapping epitopes required for the interaction of p75^NTR^ DD with both RIP2 and RhoGDI (Charalampopoulos et al., 2012). In order to better understand the hierarchical relationship of these interactions, we determined the binding affinities of the DD:RhoGDI and DD:CARD complexes by SPR. The *K*_d_ of RhoGDI binding to the p75^NTR^ DD was 0.82 ± 0.3 μM, while the *K*_d_ of CARD binding to the DD was 4.67 ± 0.7 nM (Figure 4B,C), indicating that RIP2 CARD binds with approximately 177-fold higher affinity than RhoGDI to the p75^NTR^ DD. This is in line with the larger buried solvent accessible area in the p75^NTR^ DD:RIP2 CARD complex (~1400 Å^2^) compared to that in the p75^NTR^ DD:RhoGDI complex (~900 Å^2^). Kinetic analyses revealed that CARD associates with the p75^NTR^ DD with faster on-rate, and dissociates with slower off-rate, than RhoGDI (Table 3). The RIP2 CARD could still fold into a typical α-helical structure after deletion of the C-terminal tail (Figure 3—figure supplement 4A). However, the binding affinity of this construct to the p75^NTR^ DD was 58.7 ± 8.8 nM, that is, approximately 12-fold lower than with the C-terminal tail (Figure 3—figure supplement 4B), indicating a significant contribution of the C-terminal tail to the association of RIP2 with p75^NTR^.

**Table 3.** Association and dissociation binding constants of p75NTR DD binding to RhoGDI and RIP2 CARD. **DOI:** http://dx.doi.org/10.7554/eLife.11692.016

	ka (µM^-1^·s^-1^)	kd (s^-1^·10^-3^)	Kd (nM)
DD:RhoGDI	0.06 ± 0.01	50 ± 9	827 ± 338
DD:CARD	0.72 ± 0.34	3.32 ± 1.5	4.67 ± 0.7

The differential binding of RhoGDI and RIP2 CARD to the p75^NTR^ DD was further tested through analysis of 2D NMR spectra of RhoGDI binding to p75^NTR^ DD in competition with RIP2 CARD. Figure 4D shows details of the RhoGDI spectra focusing on representative residues located in and/or close to the DD:RhoGDI interface. Addition of p75^NTR^ DD produced a shift in the cross-peaks of these residues (red in Figure 4D), indicating binding of RhoGDI to the DD. Addition of RIP2 CARD to the RhoGDI:DD complex shifted these cross-peaks back to their initial positions (arrows in Figure 4D), indicating dissociation of RhoGDI from the p75^NTR^ DD. These data demonstrate that RhoGDI and RIP2 CARD compete for binding to the p75^NTR^ DD and that RIP2 CARD can displace RhoGDI from the receptor. In order to test the functional significance of the antagonism between RIP2 and RhoGDI, we assessed the levels of RhoA-GTP, a measure of RhoA activation, in cells transfected with a p75^NTR^ expression construct in the absence or presence of increasing concentrations of a RIP2 construct (Figure 4E). While expression of p75^NTR^ increased RhoA-GTP levels in transfected cells, coexpression of RIP2 decreased RhoA-GTP levels in a concentration-dependent manner (Figure 4E), in agreement with an inhibitory role of RIP2 in p75^NTR^-mediated activation of the RhoA pathway. In line with this, we found elevated levels of RhoA-GTP in brain extracts from *Rip2* knockout mice compared to wild type littermates (Figure 4F), suggesting that RIP2 can also restrict the activation of the RhoA pathway in vivo.

### Solution structure of the p75^NTR^ DD homodimer

The current model of p75^NTR^ activation by neurotrophins predicts that the DDs should be in close proximity to each other to account for the high FRET state of the unliganded receptor. Purified rat p75NTR DD has been shown to exist in either monomeric form or equilibrium between monomeric and dimeric forms depending on pH and counterion (Vilar et al., 2014). However, the complete assignment of the DD homodimer was not reported in that study. We also found that human p75^NTR^ DD exists mainly in monomeric form in TRIS or HEPES buffer at pH 6.0–7.0, which were the buffer conditions used for structure determination of DD:RhoGDI and DD:CARD complexes. In phosphate buffer, however, we observed a new form of p75^NTR^ DD as revealed by the appearance of a new set of cross peaks in the [^1^H-^15^N] HSQC spectrum (Figure 5—figure supplement 1A,B). The set of cross peaks corresponding to monomeric DD was still visible, with nearly identical chemical shift but weaker intensity (Figure 5—figure supplement 1A), suggesting the coexistence of dimeric and monomeric DDs under these conditions. Dynamic lighter scattering (DLS) also indicated the formation of dimeric p75^NTR^ DDs in the presence of phosphate ions (Figure 5—figure supplement 1C,D). EGFP-tagged p75^NTR^ DDs showed anisotropic changes due to homodimerization at different DD concentrations. The apparent *K*_d_ of dimerization derived from anisotropic change was 49 ± 15 µM (Figure 5—figure supplement 1E). This relatively low-affinity interaction may facilitate DD separation (low FRET state) upon receptor activation by neurotrophins.

**Figure 5.**
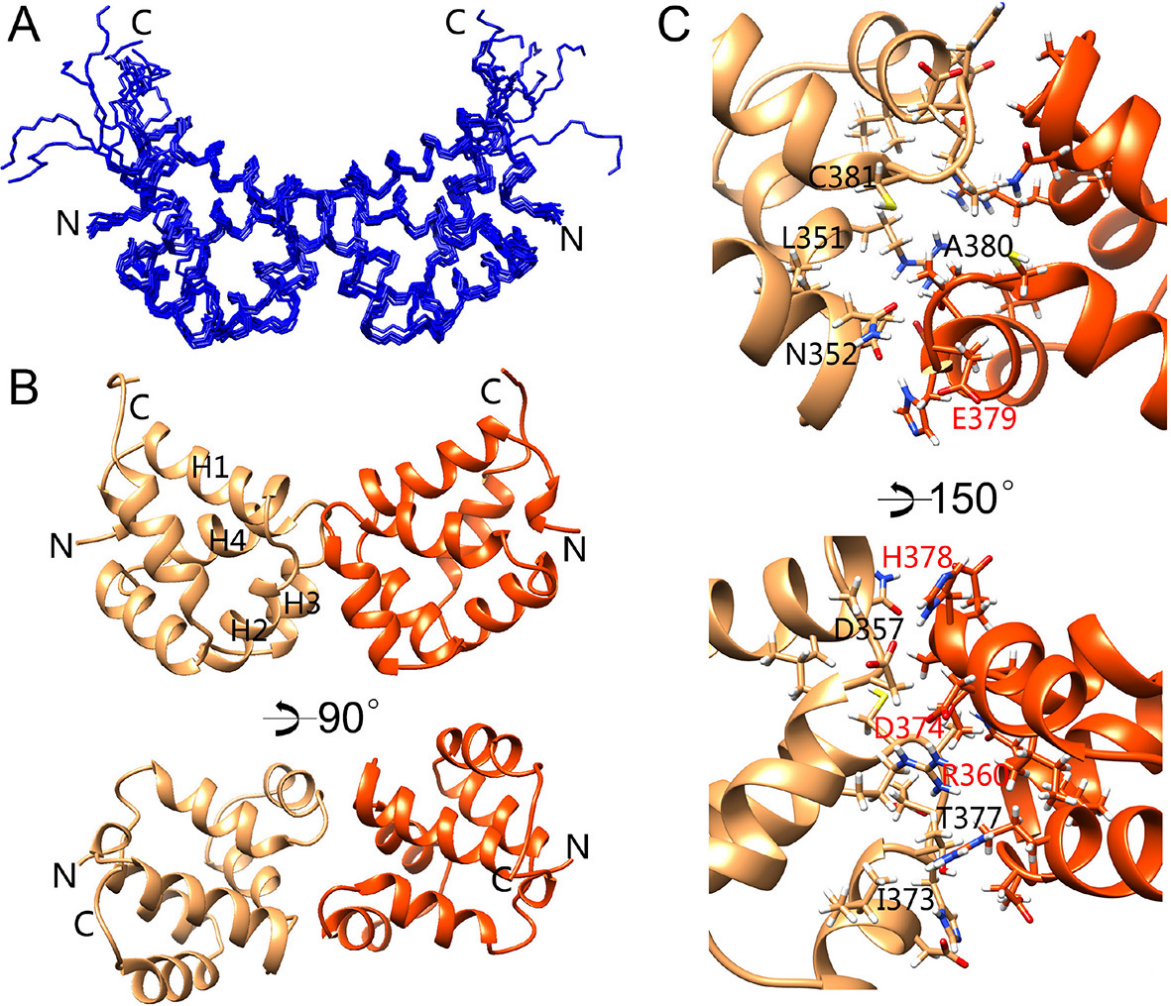
Solution structure of the p75^NTR^ DD homodimer. (**A**) Superposition of backbone heavy atoms of the 10 lowest-energy structures of the human p75^NTR^ DD homodimer. N- and C-termini are indicated. (**B**) Ribbon drawing of the lowest-energy conformer viewed perpendicular (top) and parallel (bottom) to the twofold symmetry axis. DD monomers are colored in light brown and orange. N- and C-termini, as well as DD helices H1, H2, H3, and H4 are indicated. (**C**) Detail of binding interface in the DD homodimer. The top image shows the same view as that in panel B, bottom. Key residues at the binding interface are labeled and depicted as stick models. Red labels denote interface residues functionally validated in a previous mutagenesis study (Vilar et al., 2014). **DOI:** http://dx.doi.org/10.7554/eLife.11692.017

In order to identify the dimerization interface, we determined the NMR structure of the p75^NTR^ DD homodimer. The p75^NTR^ DD homodimer adopted a C2 symmetry (Figure 5A,B). A short C-terminal tail of 7 amino acids (Ser^421^-Val^427^) in each monomer remained disordered, similar to the DD:RhoGDI and DD:CARD complexes. The helical bundle, including the 3_10_ helix, did not undergo significant structural change with an RMSD lower than 1.5 Å compared to the other complexes. The dimerization interface consisted of α-helices H2 and H3 as well as residues in the H1–H2 and H3–H4 loops. Dimerization involved both charge and hydrophobic interactions. The key residues in the dimer interface included Asp^357^, Arg^360^, Thr^377^, His^378^, Glu^379^, and Ala^380^ (corresponding to Asp^354^, Arg^358^, Thr^375^, His^376^, Glu^377^, and Ala^378^ in rat p75^NTR^ DD). This is in agreement with previous site-directed mutagenesis and NMR titration studies of the rat p75^NTR^ DD homodimer (Vilar et al., 2014) (red-labeled residues in Figure 5C). Cys^381^ (homologous to Cys^379^ in rat p75^NTR^ DD) was also located in the dimerization interface but appeared in reduced form with a C^β^ chemical shift of 26.5 ppm. The distance between Cys^381^S^γ^ from each monomer was 6.7 ± 0.1 Å, that is, too long for the formation of a disulfide bond. The buried solvent accessible area in the p75^NTR^ DD homodimer was 573 Å^2^, in line with the low affinity of the DD:DD interaction. We conclude that the two p75^NTR^ DD protomers form a low-affinity, noncovalent homodimer in our structure.

**Figure 5—figure supplement 1.**
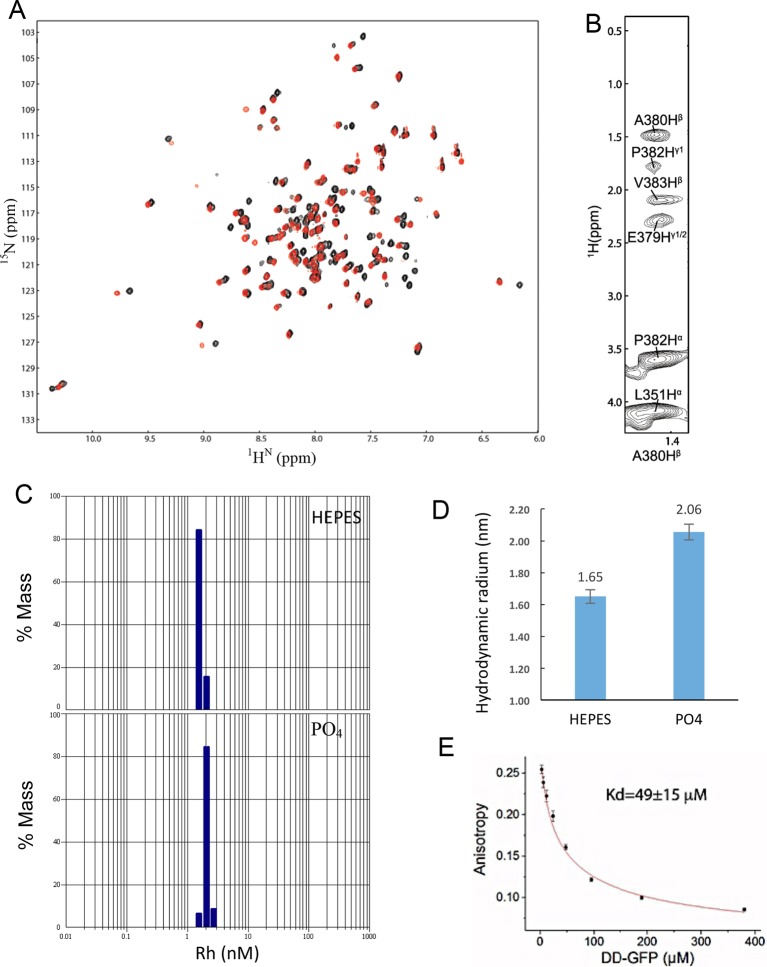
Homodimerization of p75^NTR^ DD. (**A**) [^1^H-^15^N] HSQC spectra of p75^NTR^ DD in HEPES buffer (black) and phosphate buffer (red) at pH 6.9 and 28°C. (**B**) Representative slices from the ^13^C,^15^N-filtered 3D NOESY spectrum. (**C**) Apparent hydrodynamic radius (Rh) distribution of DD from DLS measurement in HEPES (top) and phosphate buffer (bottom), respectively. The protein concentrations used are ~0.2 mM. (**D**) Average Rh of DD in HEPES and phosphate buffers. The theoretical Rh of DD monomer (~10 kDa) and homodimer (~20 kDa) are ~1.6 and ~2.2 nm, respectively. (**E**) Determination of monomer-dimer *K*_d_ using anisotropy change resulting from FRET. p75^NTR^ DD tagged with EGFP(A206K) in its C-terminus was used in these experiments. *N*= 3. **DOI:** http://dx.doi.org/10.7554/eLife.11692.018

**Figure 6.**
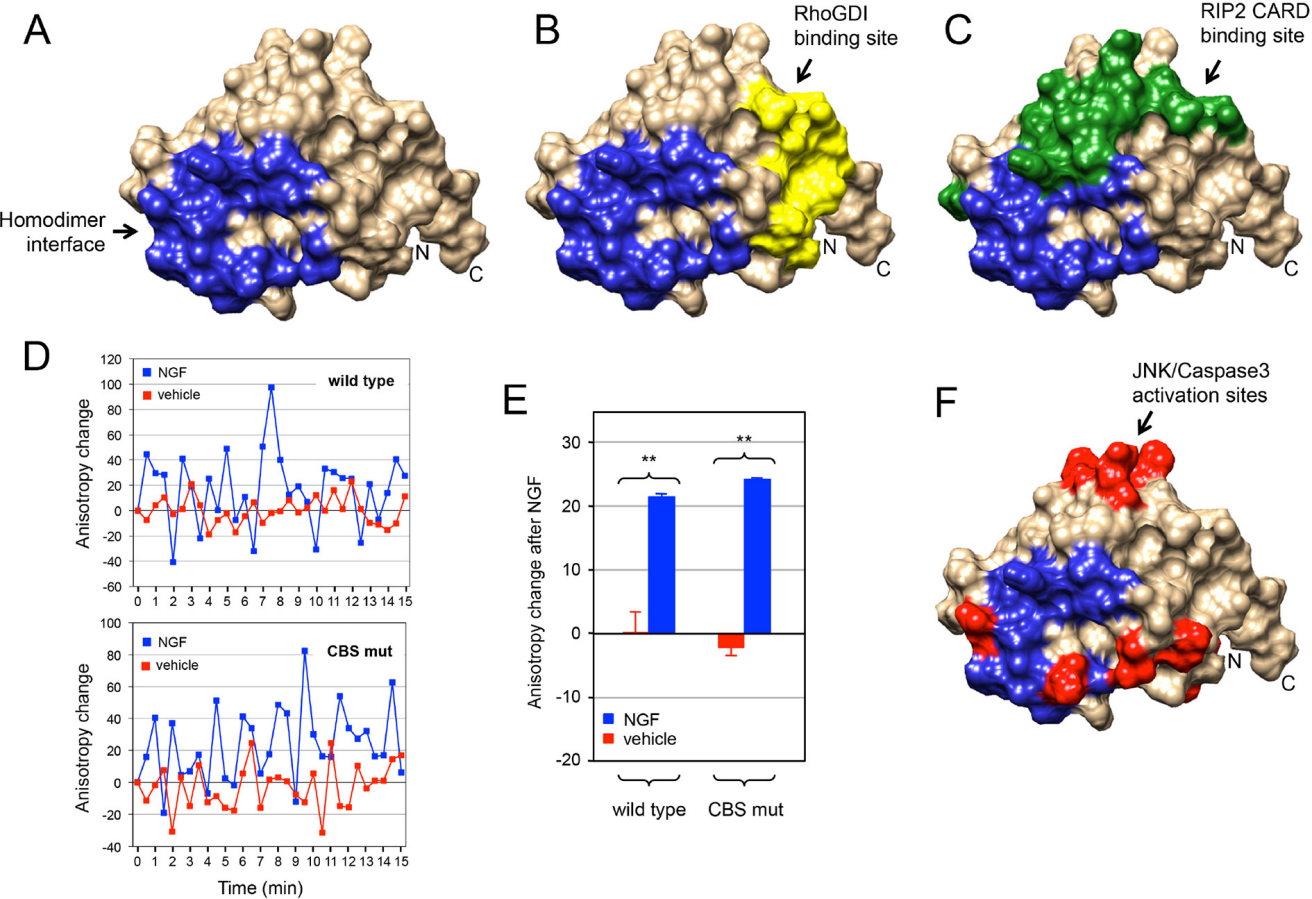
Relationship between p75^NTR^ DD dimer interface and sites of interaction with downstream effectors. (**A**) Surface presentation of p75^NTR^ DD with homodimer interface colored in blue. N- and C-termini are indicated. (**B, C** and **F**) Representation of RhoGDI binding site (yellow in (B) RIP2 CARD binding site (green in (C) and JNK/caspase-3 activation sites (from [Charalampopoulos et al., 2012]) (red in (**F**) on the p75^NTR^ DD surface showing overlap of DD homodimer interface (blue) with CARD binding and JNK/caspase-3 activation sites but not with RhoGDI binding site. N- and C-termini are indicated. (**D**) Representative experiment showing traces of average anisotropy change after addition of NGF or vehicle in cells expressing wild type p75^NTR^ or a CARD binding site mutant (CBS mut) that is unable to bind RIP2 (Charalampopoulos et al., 2012). Addition of NGF, but not vehicle, induced positive anisotropy oscillations above baseline (horizontal axis at 0) in both wild type and mutant receptor constructs. (**E**) Net anisotropy change over 15 min after addition of NGF or vehicle in cells expression wild type p75^NTR^ or the CARD binding site mutant (CBS mut). Results are expressed as average ± SD (N = 3 experiments; *n *= 15–17 cells examined per experiment). **p < 0.001 vs. vehicle. **DOI:** http://dx.doi.org/10.7554/eLife.11692.019

The p75^NTR^ DD homodimer interface defined in our NMR studies did not overlap with the RhoGDI binding site (Figure 6A,B). This is in agreement with the constitutive interaction of RhoGDI with the closed conformation of the receptor. On the other hand, the DD homodimer partially occluded the RIP2 CARD interaction surface (Figure 6C), indicating that interaction of RIP2 with p75^NTR^ requires dissociation of the DD homodimer. We investigated whether the recruitment of RIP2 contributes to the separation of DDs triggered after neurotrophin binding to the receptor. This was done by taking real-time homo-FRET anisotropy measurements of DD:DD interaction in response to NGF in cells transfected with EGFP-tagged constructs of full length wild type p75^NTR^ and a DD mutant deficient in RIP2 binding (Charalampopoulos et al., 2012) as previously described (Vilar et al., 2009). Application of NGF to cells expressing wild type p75^NTR^ produced large oscillations of increased anisotropy at the cell membrane (Figure 6D), resulting in a positive net change averaged over a 15-min treatment compared to vehicle (Figure 6E). As anisotropy is inversely related to FRET, this behavior indicates ligand-triggered separation of receptor intracellular domains, as proposed earlier (Vilar et al., 2009). We note that the oscillations observed here are unlikely to represent the synchronous behavior of ensembles of receptors, as their average period (2–3 min) would seem too slow to reflect real molecular dynamics. Importantly, the p75^NTR^ construct carrying mutations in the CARD binding site (CBS mutant) produced very similar anisotropy changes after NGF treatment (Figure 6D,E), indicating that recruitment of RIP2 is not required for DD separation in response to ligand binding to p75^NTR^. Finally, we note that the DD homodimer interface also overlapped with several residues involved in neurotrophin-mediated activation of JNK, caspase-3 and the apoptosis pathway (Figure 6F) as identified in our previous studies (Charalampopoulos et al., 2012). Thus, while the structure and biochemical properties of the p75^NTR^ DD homodimer support the ligand-independent interaction of RhoGDI with the receptor, they also demonstrate that dissociation of the p75^NTR^ DD homodimer is required for recruitment of RIP2 and for activation of the JNK/caspase-3 pathway in response to neurotrophins.

## Discussion

### Novel heterotypic interactions in the death domain superfamily

The main paradigm in signal transduction by DD-containing proteins is oligomerization via homotypic DD interactions. Although p75^NTR^ contains a DD, which is required for downstream signaling, no intracellular p75^NTR^ effectors containing canonical DDs have been identified. The study of DD signaling in p75^NTR^ therefore addresses an unexplored dimension of the repertoire of interactions and activities in the DD superfamily. Homotypic interactions between DDs have been classified as type I, II, and III according to the interfaces involved (Park, 2011; Park et al., 2007b; Weber and Vincenz, 2001). Despite what might have been expected of homotypic interactions, all known interactions in the DD superfamily are asymmetric, that is, the interaction is mediated by different interfaces in each of the two interacting domains (e.g., Ia and Ib for type I). Remarkably, none of the binding surfaces in the p75^NTR^ DD (or in RIP2 CARD) identified in this study show a close match to any of the six conserved surfaces that characterize classical type I, II, and III interactions in the DD superfamily. The p75^NTR^ DD surface that binds RhoGDI is formed by residues in helices H1 and H6. A previous study had proposed helix 5 as a binding site to RhoGDI based on serial deletion analysis of the p75^NTR^ DD (Yamashita and Tohyama, 2003). This conclusion is not supported by our solution structure of the DD:RhoGDI complex, in which H5 appears at the opposite side of the interface (Figure 2B), nor by previous structure–function studies (Charalampopoulos et al., 2012). This discrepancy highlights some of the pitfalls in serial deletion studies that disregard the three-dimensional structures of proteins. The surface in the p75^NTR^ DD that interacts with the CARD of RIP2 includes residues in helices H2, H3, and H6 plus residues in the H5–H6 loop. On the other side of this interaction, residues in helix H1 as well as the H3–H4 and H5–H6 loops form the binding surface on the CARD of RIP2. To the best of our knowledge, this p75^NTR^ DD:RIP2 CARD complex represents the first structural characterization of an heterotypic interaction in the DD superfamily. Our solution structure of this complex also revealed an additional interaction between residues in the C-terminal half of helix H5 of the p75^NTR^ DD and the C-terminal tail of RIP2, which extends beyond the RIP2 CARD. This additional contact confers approximately fivefold increased binding affinity between the two proteins. Finally, the interface that mediates the p75^NTR^ DD homodimer involves residues located in helix H3 as well as the H1–H2 and H3–H4 loops. This surface is similar, but not identical, to the type IIIb surface, like the one identified in the DD of PIDD for interaction with RAIDD (Park et al., 2007b). Unlike the classical type IIIb surface, however, the DD:DD interaction in p75^NTR^ makes extensive use of residues in the H3 helix, and the same surface in the two interacting DDs is used to form a symmetric dimer. Further studies will be required to determine whether the interactions identified here for the p75^NTR^ DD are exceptions or else represent new types of interactions that are yet to be identified in other DD-containing proteins.

### A symmetric DD homodimer in p75^NTR^

The solution structure of the p75^NTR^ DD homodimer shows it is a symmetric, noncovalent dimer held together by low-affinity interactions involving residues in helix H3 and the H1–H2 and H3–H4 loops. The p75^NTR^ DD dimer interface is in agreement with sites of interaction with downstream effectors identified by the structures reported here and in our previous site-directed mutagenesis studies (Figure 6). This p75^NTR^ DD dimer structure is also in accordance with a recent mutagenesis study that identified residues important for dimerization of rat p75^NTR^ DD (Vilar et al., 2014), many of which are also implicated in our structure. On the other hand, our results do not support two crystallographic structures reported for the rat p75^NTR^ DD homodimer that suggested this to be either a covalent symmetric dimer, held by a disulfide bond between Cys^379^ residues, or a noncovalent asymmetric dimer (Qu et al., 2013). None of the currently available evidence derived from structural, mutagenesis, or functional studies appears to support those crystal structures. Nevertheless, we cannot at present rule out the possibility that p75^NTR^ DDs may under certain circumstances form alternative oligomeric complexes through different interfaces. A recent study has suggested that p75^NTR^ can form trimers in transfected cells based on the apparent molecular weights of p75^NTR^ species in SDS/PAGE (Anastasia et al., 2015). Our NMR studies of the p75^NTR^ DD do not support such conclusion as we have not found any evidence for the existence of DD trimers in any of the conditions tested. Another recent study has used solution NMR spectroscopy to investigate the mobility of the transmembrane and intracellular domains of p75^NTR^ incorporated into lipid–protein nanodisks (Mineev et al., 2015). These authors found a high level of flexibility in the juxtamembrane domain of p75^NTR^, an observation that we also reported in our earlier NMR studies of this domain (Liepinsh, 1997), but they could not detect self-association of intracellular domains. However, it is unclear whether the lipid detergent used to form the lipid–protein nanodisks interacted with the p75^NTR^ DD and prevented its dimerization. A few detergent molecules, too few to affect DD rotational correlation time, would be sufficient to hinder DD:DD interactions.

### Competitive protein–protein interactions define the hierarchical activation of downstream pathways in p75^NTR^ signaling

The mechanism underlying ligand-induced dissociation of RhoGDI from p75^NTR^ has remained unclear. As neurotrophins induce the release of RhoGDI and the recruitment of RIP2, we have speculated that RIP2 may displace RhoGDI from binding sites in the p75^NTR^ DD (Charalampopoulos et al., 2012). Our solution structures of the p75^NTR^ DD in complex with RhoGDI and the RIP2 CARD lend experimental support to this notion by showing how steric clashes between the two effector proteins preclude their simultaneous binding to the p75^NTR^ DD. SPR experiments indicated that RIP2 CARD binds with over 100-fold higher affinity to the p75^NTR^ DD than RhoGDI, and 2D-NMR competition studies demonstrated that RIP2 CARD can in fact displace RhoGDI from the receptor. The functional significance of this relationship was evidenced by the ability of RIP2 to decrease p75^NTR^-mediated RhoA activation in a dose-dependent manner. Furthermore, the enhanced activation of the RhoA pathway observed in brain extracts of *Rip2* knockout mice suggests that RIP2 may also restrict activation of this pathway in vivo. These results demonstrate how a hierarchy of binding affinities dictates the differential interaction of downstream effectors with p75^NTR^ and ultimately controls the logic of p75^NTR^ signaling.

### A model for the early stages of p75^NTR^ engagement with the RhoA and NF-kB pathways

**Figure 7.**
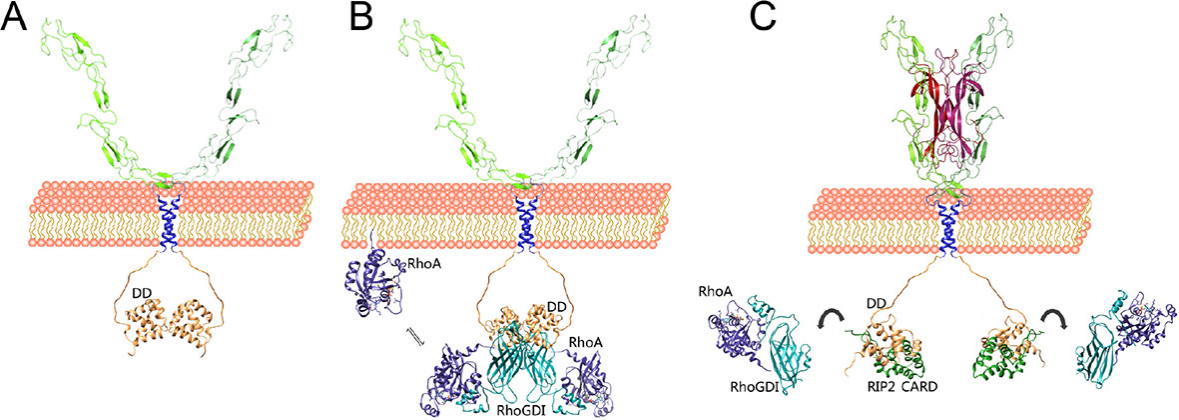
Competitive protein–protein interactions orchestrate coupling of p75**^NTR^** to the RhoGDI/RhoA and RIP2/NF-kB pathways. Schematic drawing of a model for the coupling of p75^NTR^ to the RhoGDI/RhoA and RIP2/NF-kB pathways based on the structural and biochemical studies presented above. (**A**) The p75^NTR^ dimer in the cell membrane is held by homotypic interactions of DDs (light brown) and TM domains (blue). (**B**) RhoGDI (cyan) brings RhoA (dark purple) in proximity to the plasma membrane through its interaction with the DD of p75^NTR^. While the twofold symmetry axis of the DD:RhoGDI:RhoA hexametric complex is likely to be perpendicular to the plasma membrane, its relative orientation is hypothetical. RhoGDI binding to the p75^NTR^ DD decreases its affinity for RhoA by 15-fold, and allows equilibration of RhoA with the plasma membrane, where it can be activated by GEFs. (**C**) Neurotrophin binding induces a conformational change in p75^NTR^ resulting in the separation of its DDs (Vilar et al., 2009), exposing binding sites to downstream effectors that couple to the JNK/caspase-3 or NF-kB pathways, including RIP2. Recruitment of RIP2 to the p75^NTR^ DD is mediated by the interaction of its CARD (green) with a binding surface that partially overlaps with that occupied by RhoGDI. As the binding affinity of the RIP2 CARD for the p75^NTR^ DD is 100-fold higher than that of RhoGDI, the recruitment of RIP2 displaces RhoGDI from the receptor. Released from the DD, RhoGDI regains higher affinity for RhoA, extracting it from the membrane and holding it back in the cytosol. **DOI:** http://dx.doi.org/10.7554/eLife.11692.021

p75^NTR^ has been postulated to function as a “displacement factor” that releases RhoA from RhoGDI leading to RhoA activation (Yamashita and Tohyama, 2003). This model has led to the idea that the p75^NTR^ DD and RhoA may compete for binding to RhoGDI. On the other hand, biochemical experiments have shown that RhoA can associate with p75^NTR^ through RhoGDI and the three proteins can be recovered together in co-immunoprecipitation assays (Yamashita et al., 1999; Yamashita and Tohyama, 2003), a result that would be incompatible with the displacement concept. Our structural studies show that the p75^NTR^ DD and RhoA bind on opposites sides of the RhoGDI molecule, allowing the formation of a tripartite DD:RhoGDI:RhoA complex. Using a model of this complex and our solution structure of the p75^NTR^ DD homodimer, we have constructed a model of the hexameric complex of these proteins (Video 1). This model retains the two fold symmetry of the DD homodimer, and accommodates all six components without any steric clashes. How can these interactions lead to RhoA activation? Our SPR experiments showed that association of RhoGDI with the p75^NTR^ DD reduced its affinity for RhoA by 15-fold. Close comparison of RhoGDI structures in the complexes with either p75^NTR^ DD or RhoA revealed local structural perturbations in RhoGDI (Figure 2—figure supplement 1), suggesting a potential allosteric mechanism underlying the release and activation of RhoA upon RhoGDI biding to the receptor. Based on the present studies, we propose a model for the early stages of p75^NTR^ engagement with the RhoGDI/RhoA and RIP2/NF-kB pathways based on differential binding affinities and competitive protein–protein interactions (Figure 7). At the plasma membrane, the p75^NTR^ forms a dimer held together by interactions between its DD and TM domains (Figure 7A). Recruitment of the RhoGDI:RhoA complex brings RhoA close to the plasma membrane (Figure 7B). RhoGDI binding to the p75^NTR^ DD weakens its interaction with RhoA, a lipid-modified protein, allowing it to equilibrate with the plasma membrane where it can be activated by membrane-associated guanine nucleotide exchange factors (GEFs) (Garcia-Mata et al., 2011). RhoA may thus be in equilibrium between the cell membrane and the RhoGDI:p75^NTR^ complex, and the action of GEFs and GTPase-activating proteins (GAPs) may further contribute to the dynamics of this exchange (Figure 7B). Upon dissociation from p75^NTR^, for example, as a consequence of RIP2 recruitment in response to NGF binding, RhoGDI regains high affinity for RhoA, extracting it from the membrane and holding it back in the cytosol in an inactive state (Figure 7C). This new view of the p75^NTR^ DD in the activation of RhoA is in better agreement with the emerging role of RhoGDI as a general facilitator of small GTPase activity cycles. Recruitment of RIP2 to p75^NTR^ ultimately leads to increased NF-kB activity by as yet unknown mechanisms. Another p75^NTR^ interactor, TRAF6, is also a known regulator of the NF-kB pathway (Khursigara et al., 1999; Ye et al., 1999). As TRAF6 has been shown to interact with the juxtamembrane region of p75^NTR^, but not with the DD, RIP2 and TRAF6 may be able to bind simultaneously to the receptor and together contribute to enhance NF-kB activity in response to neurotrophins.

**Video 1.** Model of the hexameric complex between p75^NTR^, RhoGDI and RhoA(GDP). Animation around the two-fold symmetry axis of the hexameric p75^NTR^ DD:RhoGDI:RhoA(GDP) complex. p75^NTR^ DD appears in light brown, RhoGDI in cyan and RhoA in blue. **DOI:** http://dx.doi.org/10.7554/eLife.11692.020

### Conclusions

The structural studies of DD signaling in p75^NTR^ presented here uncovered novel heterotypic interactions not previously seen in other DD-containing complexes. They represent new ways by which DDs regulate intracellular signaling. NMR, biochemical, and functional studies defined competitive interactions between RhoGDI and RIP2 CARD and between RIP2 CARD and the p75^NTR^ DD homodimer. These interactions give us unique insights into the molecular mechanisms underlying p75^NTR^ activation and signaling, and reveal how overlapping interfaces and differential binding affinities cooperate to orchestrate the hierarchical activation of downstream pathways in noncatalytic receptors.

## Materials and methods

### Sample preparation

The cDNAs of human p75^NTR^ DD (330–427), RhoGDI (2–204), RhoA (2–190), and RIP2 CARD (434–539) were amplified from total human embryonic stem (ES) cell cDNA and subcloned into pET32-derived expression vectors between BamH I and Xho I restriction sites. Each recombinant protein contains 16 additional residues (MHHHHHHSSGLVPRGS) at the N-terminal, including one 6×His tag. Unlabeled proteins were expressed in *E. coli* strain SoluBL21 (DE3) in LB or M9 medium. Protein samples were purified using Ni-NTA affinity chromatography, FPLC gel filtration (Superdex 75), and/or ionic exchange (MonoQ or MonoS). Isotopic labeling was carried out by expressing the proteins in M9 minimal medium containing ^15^N-NH_4_Cl and/or ^13^C-labeled glucose as the sole source of nitrogen and carbon. Protein complexes were prepared by mixing individual purified domains. Due to the weak binding affinities of DD:RhoGDI and DD homodimer complexes, as well as solubility problems of the DD:CARD complex in salt-containing buffers, gel filtration chromatography could not be used to purify these protein complexes. For the p75^NTR^ DD:RhoGDI complex, two double-labeled samples were prepared in 10 mM D18-HEPES, 10 mM D10-DTT, 1 mM EDTA, and 0.01% sodium azide at pH 6.9: (1) 0.5 mM ^13^C,^15^N-labeled p75^NTR^ DD mixed with 2 mM unlabeled RhoGDI; (2) 0.5 mM ^13^C, ^15^N-labeled RhoGDI mixed with 2 mM unlabeled p75^NTR^ DD. For the p75^NTR^ DD:RIP2 CARD complex, two double-labeled samples were made in water with 10 mM D10-DTT: (1) 0.5 mM ^13^C, ^15^N-labeled p75^NTR^ DD mixed with 1 mM unlabeled RIP2 CARD; (2) 0.5 mM ^13^C, ^15^N-labeled RIP2 CARD mixed with 1 mM unlabeled p75^NTR^ DD. For the RIP2 CARD on its own, 0.7 mM ^13^C, ^15^N-labeled RIP2 CARD was prepared in 50 mM D10-DTT in water. For the p75^NTR^ DD homodimer, 1 mM ^13^C, ^15^N-labeled p75^NTR^ DD was mixed with 1 mM unlabeled p75^NTR^ DD in 50 mM phosphate buffer, 10 mM D10-DTT, 1 mM EDTA and 0.01% sodium azide at pH 6.9.

### NMR spectroscopy and structure calculations

NMR experiments were performed on a Bruker 800 MHz NMR spectrometer with a cryogenic probe at 28°C. All spectra were processed with NMRPipe (Delaglio et al., 1995) and analyzed with NMRView supported by a NOE assignment plugin (Johnson and Blevins, 1994). Resonance assignments of backbone, aliphatic, and aromatic side chains were obtained using previously described methods (Lin et al., 2006; Xu et al., 2006). Intramolecular NOE restraints were obtained from 4D time-shared ^13^C, ^15^N-edited NOESY spectra (Xu et al., 2007). Intermolecular NOEs were identified from ^13^C,^15^N-filtered 3D experiments (Zwahlen et al., 1997). Ambiguous NOEs were assigned with iterated structure calculations by DYANA (Herrmann et al., 2002). Final structure calculation was started from 100 conformers. Energy minimization of the 10 conformers with the lowest final target function values was performed in AMBER force field (Case et al., 2002). The mean structure was obtained from the 10 energy-minimized conformers for each domain. PROCHECK-NMR (Laskowski et al., 1996) was used to assess the quality of the structures. All the structural figures were made using MOLMOL (Koradi et al., 1996) or Chimera (Pettersen et al., 2004). The coordinates of p75^NTR^ DD:RhoGDI, RIP2 CARD, p75^NTR^ DD:RIP2 CARD, and p75^NTR^ DD homodimer have been deposited with the Protein Data Bank with PDB IDs 2n80, 2n7z, 2n83, and 2n97, respectively.

### Model structure calculations, structure-based alignments and structural comparison

The structure of DD:RhoGDI:RhoA was modeled using HADDOCK 2.2 (Dominguez et al., 2003). The starting structures for the trimer were the DD monomeric structure from the lowest-energy structure of p75^NTR^ DD:RhoGDI complex and the crystal structure of human RhoGDI:RhoA(GDP) (PDB ID: 1CC0). The starting structures used to build the hexameric model were the lowest-energy structure of the p75^NTR^ DD homodimer and the crystal structure of human RhoGDI:RhoA(GDP). NOE data between p75^NTR^ DD and RhoGDI were employed to create interaction restraints for both trimer and hexamer. Totally, 1000 rigid-body docking solutions were first generated by energy minimization. The best 100 structures according to intermolecular energies were subjected to semi-flexible simulated annealing in torsion angle space followed by a final refinement in explicit water. Pairwise structure-based alignment and comparison were carried out using a sequential structure alignment program (SSAP) available at the SSAP server (http://www.cathdb.info/cgi-bin/cath/SsapServer.pl).

### Dynamic lighter scattering (DLS) and circular dichroism (CD)

The apparent hydrodynamic radii of p75^NTR^ DD domain in HEPES or phosphate buffer at pH 7.0 were examined by DLS (DynaPro, Protein Solutions Inc., Lakewood, NJ) at 22°C. The data were analyzed using Dynamics 5.0 software. The CD spectra of all samples were recorded on a Jasco J-810 spectropolarimeter equipped with a thermal controller at 22°C.

### Surface plasmon resonance (SPR) and anisotropy measurements of DD homodimerization

All sensorgrams were recorded on a BIAcore T200 at 22°C. For experiments with captured p75^NTR^ DD (ligand), purified p75^NTR^ DD-His was captured onto NTA sensor chips via Ni2+/NTA chelation. Protein samples of purified RIP2 CARD or RhoGDI (analytes) were sequentially diluted in running buffer (10 mM HEPES, 50 mM NaCl, 0.005% Surfactant P20, 0.02% protease-free BSA at pH 7.0) and injected over the surfaces at different concentrations post capture. Regeneration of the NTA surface was performed using 350 mM EDTA. For experiments involving immobilized RhoA, unprenylated RhoA:GDP:Mg2+ was immobilized via amine coupling onto CM5 sensor chips. Unreacted carboxymethyl sites were capped by ethanolamine. Protein samples of analytes were sequentially diluted in running buffer (10 mM HEPES, 50 mM NaCl, 0.005% Surfactant P20, pH 7.0) and injected over the surfaces at different concentrations. To measure the binding of RhoGDI to RhoA, 1 mM MgCl2 and 100 µM GDP were also included in the running buffer. Binding affinities were expressed as equilibrium dissociation constants (*K*_d_) determined by steady state (Figure 2C,D) or kinetic analyses (Figures 4B,C and Figure 3—figure supplement 4B) using the BIA evaluation software. One binding site model was used for fitting of SPR data.

For anisotropy measurements of DD homodimerization, a cDNA encoding Enhanced Green Fluorescent Protein (EGFP) carrying the A206K mutation (to prevent its dimerization) was linked to the C-terminal of the human p75^NTR^ cDNA via DNA ligation and the chimera protein (p75^NTR^ DD-EGFP) was expressed in *E.* *coli* BL21(DE3) and purified by FPLC. p75^NTR^ DD-EGFP was sequentially diluted in 50 mM phosphate buffer at pH 7.0. The anisotropy value was obtained from the measurements of fluorescence intensity in both parallel and perpendicular emission modes using a BioTek Cytation Imaging Reader at room temperature. Dimer dissociation constants were obtained by nonlinear fitting of anisotropy measurements to an equation describing a monomer–dimer equilibrium (Martin and Martin, 1996).

### Plasmids, antibodies, and mice

Full-length cDNAs encoding human p75^NTR^, RhoGDI and RIP2 were amplified from human embryonic stem (ES) cell cDNA and subcloned in the pCDNA3 vector backbone (Invitrogen) for protein expression in mammalian cells. Mutations and epitope tags were introduced using QuickChange Site-Directed Mutagenesis Kit (Stratagene, United Kingdom) and verified by DNA sequencing. Normal expression of all constructs was verified by immunoblotting. The origin of antibodies was as follows: ANT-007 anti-p75^NTR^ (for immunoprecipitation) from Alomone Labs; ab52987 anti-p75^NTR^ (for immunoblotting) and anti-RhoGDI from Abcam; anti-Myc from Cell Signaling Technologies; anti-RIP2 from Enzo Life Sciences; anti-β-actin and anti-βIII-tubulin from Sigma-Aldrich. Rip2 knockout mice were obtained from Koichi Kobayashi and Richard Flavell (Kobayashi et al., 2002).

### Cell culture, cell transfection, immunoprecipitation, immunoblotting, and RhoA activation assay

HEK293 and COS-7 cells were obtained from ATCC and cultured under standard conditions in DMEM supplemented with 10% fetal calf serum, 100 units/ml penicillin, 100 mg/ml streptomycin, and 2.5 mM glutamine. HEK293 cells were transfected with the polyethylenimine (PEI) method. Briefly, cells were plated in a 10 cm tissue culture dish at a confluency of 3 × 10^6^ cells/dish in normal growth media. Twenty-four hours after plating, the media was changed to growth media containing 1% v/v FBS. Transfection mix was prepared by mixing 1 μg of plasmid with 3 μg of PEI (1 mg/ml) in DMEM. The transfection mix was left to stand at room temperature followed by addition dropwise into culture plates. 24 hrours after transfection, the transfected cells were returned to normal growth media. After a further 24 hr, cells were placed in sera-free media for 16 hr prior to harvest and lysis in 50 mM Tris/HCl pH 7.5, 1 mM EDTA, 270 mM Sucrose, 1% (v/v) Triton X-100, 1 mM benzamidine, 1 mM PMSF, 0.1% (v/v) 2-mercaptoethanol, and in the presence of phosSTOP (Roche) phosphatase inhibitor cocktail mix as per manufacturer instructions. The cellular extracts were then centrifuged at 4°C top speed on a benchtop centrifuge for 15 min. The supernatant was collected and filtered using a 0.2 μM syringe filter. Protein concentration was determined by Bradford Assay. For anisotropy microscopy, COS-7 cells were transfected with Fugene6 (Promega) according to manufacturer’s instructions. For immunoprecipitation, cell extracts (0.5 mg protein) was incubated for 16 hr at 4°C on a rotating wheel with 0.5 μg of anti-p75 antibody (ANT-007, Alomone) attached to Protein G–Sepharose (7.5 μl packed beads). The beads were collected by brief centrifugation (2 min,780× g, 4°C), washed three times with 0.5 ml of Wash Buffer (50 mM Tris/HCl pH 7.5, 1% (v/v) Triton X-100, 0.05% (v/v) 2-mercaptoethanol, and 0.2 M NaCl). After the last wash, pelleted beads were aspirated off the wash buffer followed by addition of Laemmli sample buffer and analysis by SDS-PAGE and Western Blot. Immunoblots were developed using the ECL Western Blotting Kit (Thermo Scientific) and exposed to Kodak X-Omat AR films. Image analysis and quantification of band intensities were done with ImageJ software (NIH). For RhoA activation assays, mouse cerebella were dissected from postnatal day (P) 7 pups. RhoA activity was evaluated in total cerebellar extracts or in lysates of transfected HEK293 cells using the RhoA G-Lisa kit (Cytoskeleton) following the manufacturer’s instructions. Equal amount of protein was used from each sample as determined by Bradford Assay.

### Homo-FRET anisotropy microscopy

Anisotropy microscopy was done as previously described (Vilar et al., 2009) in transiently transfected COS-7 cells. Images were acquired 24 hr post-transfection, using a Nikon Eclipse Ti-E motorized inverted microscope (Nikon, Japan) equipped with a X-Cite LED illumination system. A linear dichroic polarizer (Meadowlark Optics) was placed in the illumination path of the microscope, and two identical polarizers were placed in an external filter wheel at orientations parallel and perpendicular to the polarization of the excitation light. The fluorescence was collected via a CFI Plan Apochromat Lambda 40×, 0.95 NA air objective, and parallel and polarized emission images were acquired sequentially on an Orca CCD camera (Hamamatsu Photonics, Japan). Data acquisition was controlled by the Metamorph software (Molecular Devices, USA). NGF (from Alomone Labs) or vehicle was added 3 min after the start of the time lapse at a concentration of 100 ng/ml. Anisotropy values were extracted from image stacks of 30 images acquired in both parallel and perpendicular emission modes every 30 s for a time period of 15 min after NGF addition. For each construct, 12–15 ROIs were measured in three independent transfections performed in duplicate. Fluorescence intensity and anisotropy images were calculated as described by Squire et al. (2004). Wild type and CARD binding site (CBS) mutant cDNA constructs of rat p75^NTR^ were tagged at the C terminus with a monomeric version of EGFP (Clontech) carrying the A206K mutation that disrupts EGFP dimerization. The CBS p75^NTR^ mutant corresponded to the triple mutant D355A/H359A/E363A described in our previous study (Charalampopoulos et al., 2012).

## References

[bib1] Anastasia A, Barker PA, Chao MV, Hempstead BL (2015). Detection of p75NTR trimers: implications for receptor stoichiometry and activation. Journal of Neuroscience.

[bib2] Carter BD, Kaltschmidt C, Kaltschmidt B, Offenhauser N, Bohm-Matthaei R, Baeuerle PA, Barde Y-A (1996). Selective activation of NF-kappa b by nerve growth factor through the neurotrophin receptor p75. Science.

[bib3] Case DA, Pearlman DA, Caldwell JW, Cheatham TE, Wang III, Ross WS, Simmerling CL, Darden TA, Merz TA, Stanton, R.V KM (2002). AMBER 7.

[bib4] Charalampopoulos I, Vicario A, Pediaditakis I, Gravanis A, Simi A, Ibáñez CF (2012). Genetic dissection of neurotrophin signaling through the p75 neurotrophin receptor. Cell Reports.

[bib5] Dechant G, Barde YA (2002). The neurotrophin receptor p75(NTR): novel functions and implications for diseases of the nervous system. Nature Neuroscience.

[bib6] Delaglio F, Grzesiek S, Vuister G, Zhu G, Pfeifer J, Bax A (1995). NMRPipe: a multidimensional spectral processing system based on UNIX pipes. Journal of Biomolecular NMR.

[bib7] Dominguez C, Boelens R, Bonvin AMJJ (2003). HADDOCK: a protein−protein docking approach based on biochemical or biophysical information. Journal of the American Chemical Society.

[bib8] Feinstein E, Kimchi A, Wallach D, Boldin M, Varfolomeev E (1995). The death domain: a module shared by proteins with diverse cellular functions. Trends in Biochemical Sciences.

[bib9] Ferrao R, Wu H (2012). Helical assembly in the death domain (dD) superfamily. Current Opinion in Structural Biology.

[bib10] Friedman WJ (2000). Neurotrophins induce death of hippocampal neurons via the p75 receptor. The Journal of Neuroscience.

[bib11] Garcia-Mata R, Boulter E, Burridge K (2011). The 'invisible hand': regulation of RHO GTPases by RHOGDIs. Nature Reviews Molecular Cell Biology.

[bib12] Gehler S, Gallo G, Veien E, Letourneau PC (2004). P75 neurotrophin receptor signaling regulates growth cone filopodial dynamics through modulating RhoA activity. Journal of Neuroscience.

[bib13] Herrmann T, Güntert P, Wüthrich K (2002). Protein NMR structure determination with automated NOE assignment using the new software CANDID and the torsion angle dynamics algorithm DYANA. Journal of Molecular Biology.

[bib14] Holm L, Rosenstrom P (2010). Dali server: conservation mapping in 3D. Nucleic Acids Research.

[bib15] Ibáñez CF, Simi A (2012). P75 neurotrophin receptor signaling in nervous system injury and degeneration: paradox and opportunity. Trends in Neurosciences.

[bib16] Johnson BA, Blevins RA (1994). NMR view: a computer program for the visualization and analysis of NMR data. Journal of Biomolecular NMR.

[bib17] Khursigara G, Orlinick JR, Chao MV (1999). Association of the p75 neurotrophin receptor with TRAF6. Journal of Biological Chemistry.

[bib18] Khursigara G, Bertin J, Yano H, Moffett H, DiStefano PS, Chao MV (2001). A prosurvival function for the p75 receptor death domain mediated via the caspase recruitment domain receptor-interacting protein 2. The Journal of Neuroscience.

[bib19] Knowles JK, Rajadas J, Nguyen TV, Yang T, LeMieux MC, Vander Griend L, Ishikawa C, Massa SM, Wyss-Coray T, Longo FM (2009). The p75 neurotrophin receptor promotes amyloid-beta(1-42)-induced neuritic dystrophy in vitro and in vivo. The Journal of Neuroscience.

[bib20] Kobayashi K, Inohara N, Hernandez LD, Galán JE, Núñez G, Janeway CA, Medzhitov R, Flavell RA (2002). RICK/Rip2/CARDIAK mediates signalling for receptors of the innate and adaptive immune systems. Nature.

[bib21] Koradi R, Billeter M, Wüthrich K (1996). MOLMOL: a program for display and analysis of macromolecular structures. Journal of Molecular Graphics.

[bib22] Laskowski R, Rullmann JA, MacArthur M, Kaptein R, Thornton J (1996). AQUA and PROCHECK-NMR: programs for checking the quality of protein structures solved by NMR. Journal of Biomolecular NMR.

[bib23] Liepinsh E, Ilag LL, Otting G, Ibáñez CF (1997). NMR structure of the death domain of the p75 neurotrophin receptor. The EMBO Journal.

[bib24] Lin Z, Xu Y, Yang S, Yang D (2006). Sequence-specific assignment of aromatic resonances of Uniformly13C,15N-labeled proteins by Using13C- and15N-edited NOESY spectra. Angewandte Chemie International Edition.

[bib25] Longenecker K, Read P, Derewenda U, Dauter Z, Liu X, Garrard S, Walker L, Somlyo AV, Nakamoto RK, Somlyo AP, Derewenda ZS (1999). How RhoGDI binds rho. Acta Crystallographica Section D Biological Crystallography.

[bib26] Martin RB (1996). Comparisons of indefinite self-association models. Chemical Reviews.

[bib27] Mayle S, Boyle JP, Sekine E, Zurek B, Kufer TA, Monie TP (2014). Engagement of nucleotide-binding oligomerization domain-containing protein 1 (nOD1) by receptor-interacting protein 2 (rIP2) is insufficient for signal transduction. Journal of Biological Chemistry.

[bib28] Mineev KS, Goncharuk SA, Kuzmichev PK, Vilar M, Arseniev AS (2015). NMR dynamics of transmembrane and intracellular domains of p75NTR in lipid-protein nanodiscs. Biophysical Journal.

[bib29] Park HH, Lo Y-C, Lin S-C, Wang L, Yang JK, Wu H (2007). The death domain superfamily in intracellular signaling of apoptosis and inflammation. Annual Review of Immunology.

[bib30] Park HH, Logette E, Raunser S, Cuenin S, Walz T, Tschopp J, Wu H (2007). Death domain assembly mechanism revealed by crystal structure of the oligomeric PIDDosome core complex. Cell.

[bib31] Park KJ, Grosso CA, Aubert I, Kaplan DR, Miller FD (2010). P75NTR-dependent, myelin-mediated axonal degeneration regulates neural connectivity in the adult brain. Nature Neuroscience.

[bib32] Park HH (2011). Structural analyses of death domains and their interactions. Apoptosis.

[bib33] Perini G, Della-Bianca V, Politi V, Della Valle G, Dal-Pra I, Rossi F, Armato U (2002). Role of p75 neurotrophin receptor in the neurotoxicity by beta-amyloid peptides and synergistic effect of inflammatory cytokines. Journal of Experimental Medicine.

[bib34] Pettersen EF, Goddard TD, Huang CC, Couch GS, Greenblatt DM, Meng EC, Ferrin TE (2004). UCSF chimera?a visualization system for exploratory research and analysis. Journal of Computational Chemistry.

[bib35] Qu Q, Chen J, Wang Y, Gui W, Wang L, Fan Z, Jiang T (2013). Structural characterization of the self-association of the death domain of p75(NTR). PloS One.

[bib36] Roux PP, Barker PA (2002). Neurotrophin signaling through the p75 neurotrophin receptor. Progress in Neurobiology.

[bib37] Tnimov Z, Guo Z, Gambin Y, Nguyen UTT, Wu Y-W, Abankwa D, Stigter A, Collins BM, Waldmann H, Goody RS, Alexandrov K (2012). Quantitative analysis of prenylated RhoA interaction with its chaperone, RhoGDI. Journal of Biological Chemistry.

[bib38] Tuffereau C, Bénéjean J, Blondel D, Kieffer B, Flamand A (1998). Low-affinity nerve-growth factor receptor (p75NTR) can serve as a receptor for rabies virus. The EMBO Journal.

[bib39] Underwood CK, Coulson EJ (2008). The p75 neurotrophin receptor. The International Journal of Biochemistry & Cell Biology.

[bib40] Vicario A, Kisiswa L, Tann JY, Kelly CE, Ibanez CF (2015). Neuron-type-specific signaling by the p75NTR death receptor is regulated by differential proteolytic cleavage. Journal of Cell Science.

[bib41] Vilar M, Charalampopoulos I, Kenchappa RS, Simi A, Karaca E, Reversi A, Choi S, Bothwell M, Mingarro I, Friedman WJ, Schiavo G, Bastiaens PIH, Verveer PJ, Carter BD, Ibáñez CF (2009). Activation of the p75 neurotrophin receptor through conformational rearrangement of disulphide-linked receptor dimers. Neuron.

[bib42] Vilar M, Sung T-C, Chen Z, García-Carpio I, Fernandez EM, Xu J, Riek R, Lee K-F, Barker PA (2014). Heterodimerization of p45–p75 modulates p75 signaling: structural basis and mechanism of action. PLoS Biology.

[bib43] Wang KC, Kim JA, Sivasankaran R, Segal R, He Z (2002). P75 interacts with the nogo receptor as a co-receptor for nogo, MAG and OMgp. Nature.

[bib44] Weber CH, Vincenz C (2001). The death domain superfamily: a tale of two interfaces?. Trends in Biochemical Sciences.

[bib45] Wong ST, Henley JR, Kanning KC, Huang KH, Bothwell M, Poo MM (2002). A p75(NTR) and nogo receptor complex mediates repulsive signaling by myelin-associated glycoprotein. Nature Neuroscience.

[bib46] Xu Y, Zheng Y, Fan J-S, Yang D (2006). A new strategy for structure determination of large proteins in solution without deuteration. Nature Methods.

[bib47] Xu Y, Long D, Yang D (2007). Rapid data collection for protein structure determination by NMR spectroscopy. Journal of the American Chemical Society.

[bib48] Yamashita T, Tucker KL, Barde Y-A (1999). Neurotrophin binding to the p75 receptor modulates rho activity and axonal outgrowth. Neuron.

[bib49] Yamashita T, Tohyama M (2003). The p75 receptor acts as a displacement factor that releases rho from rho-GDI. Nature Neuroscience.

[bib50] Ye X, Mehlen P, Rabizadeh S, VanArsdale T, Zhang H, Shin H, Wang JJL, Leo E, Zapata J, Hauser CA, Reed JC, Bredesen DE (1999). TRAF family proteins interact with the common neurotrophin receptor and modulate apoptosis induction. Journal of Biological Chemistry.

[bib51] Yoon SO, Casaccia-Bonnefil P, Carter B, Chao MV (1998). Competitive signaling between TrkA and p75 nerve growth factor receptors determines cell survival. The Journal of Neuroscience.

[bib52] Zwahlen C, Legault P, Vincent SJF, Greenblatt J, Konrat R, Kay LE (1997). Methods for measurement of intermolecular NOEs by multinuclear NMR spectroscopy: application to a bacteriophage lambda N-peptide/ *boxB* RNA complex. Journal of the American Chemical Society.

