# Supplementary material for: Structural basis of death domain signaling in the p75 neurotrophin receptor
Source: eLife. 2015 Dec 8;4:e11692. doi: 10.7554/eLife.11692 (PMC4739766; doi:10.7554/eLife.11692)
Supplement: Related texts

# Article commentary: Decision letter

**DOI:** 10.7554/eLife.11692.022

**Affiliations:** Howard Hughes Medical Institute, Harvard Medical School, United States

eLife posts the editorial decision letter and author response on a selection of the published articles (subject to the approval of the authors). An edited version of the letter sent to the authors after peer review is shown, indicating the substantive concerns or comments; minor concerns are not usually shown. Reviewers have the opportunity to discuss the decision before the letter is sent (see review process). Similarly, the author response typically shows only responses to the major concerns raised by the reviewers.

Thank you for submitting your work entitled "Structural basis of death domain signaling in the p75 neurotrophin receptor" for consideration by *eLife*. Your article has been favorably evaluated by John Kuriyan (Senior editor) and three reviewers, one of whom, David Ginty, is a member of our Board of Reviewing Editors. The other two referees involved in the review process of your submission have agreed to reveal their identity: Mark Bothwell and Savvas Savvides.

The reviewers have discussed the reviews with one another and the Reviewing editor has drafted this decision to help you prepare a revised submission.

Summary

The contribution by Lin et al. uses 3D NMR to elucidate structures and death domain binding interfaces of components of p75^NTR^ signaling, including the DD domain of p75^NTR^ itself, RhoGDI coupling p75^NTR^ to RhoA activation, and RIP2 coupling p75^NTR^ to NF-kB activation. The structures are consistent with this lab's prior mutational work identifying residues that are important for these binding interactions, and are combined with biophysical and cellular studies to propose a model for p75^NTR^ signaling. Taken together with prior work from this lab, the present study offers, for the first time, a detailed and compelling model for p75^NTR^ signaling. There is substantial additional novelty in the characterization of heterotypic complexes of death domains of different families.

Essential revisions:

A major concern is that some key mechanistic conclusions in the manuscript are derived from a comparative study of binding affinities of the protein domains, and the mechanistic insights are derived from an SPR-based experimental approach that needs to be revised or at least validated in part by an orthogonal approach.

The strategy to immobilize p75^NTR^-DD and RhoA via amine coupling onto CM5 sensor chips for the binding studies via SPR introduces uncertainties about the reliability of the data obtained and the ensuing comparison of affinities. This is because the two proteins have lysine residues at or near the interaction interfaces involved in their complexes as revealed by the structural studies. Given that the coupling to CM5 sensor chips would largely occur via surface exposed lysine residues, any ensuing SPR data generated using such an approach is inherently unreliable as partial steric obstruction of available binding sites would compromise the kinetics and affinities of the interaction under study.

The authors appear to possess the necessary tools for readdressing their binding studies, given that they produced the relevant proteins for NMR studies. The Methods section implies, although it does not explicitly state, that recombinant p75^NTR^-DD and RhoA were produced as His-tagged proteins. If that is indeed the case immobilization approaches described in Wear and Wilkinshaw Analytical Biochemistry 371 (2007) 250-252 might provide a convenient alternative for at least repeating some key measurements. Otherwise, isothermal titration calorimetry might provide yet another tool for generating the necessary orthogonal information on affinities, at least for the strongest interactions in the study. Another approach, albeit experimentally more laborious, would be to produce N- or C-terminally biotinylated versions of the proteins of interest and use streptavidin biosensor chips to immobilize the proteins for SPR measurements.

Minor points (which need to be addressed in the text):

1) Why was RhoA-GDP-Mg^2+^ used for the binding studies? What are the biological extrapolations from a complex involving RhoA bound by the product of GTP hydrolysis?

2) In the first paragraph of the subsection “Solution structure of the complex between the p75^NTR^ DD and RIP2 CARD”, the authors cite a previous study reporting that the interaction between p75^NTR^ and RIP2 is mediated by their DD and CARD domains, yet, in the first paragraph of the Discussion, the authors indicate that the present study is the first heterotypic interaction described in the DD superfamily. These statements are seemingly in contradiction. Certainly the present statement is the first structural characterization of such a complex.

3) p75^NTR^ is somewhat unusual in that ligand binding terminates a constitutively active signaling pathway (RhoA). The first sentence of the Results indicates that the receptor signals through RhoA constitutively, but fails to make it clear that neurotrophin binding terminates this signaling (although they make this point later). To avoid confusing readers who lack familiarity with the p75^NTR^ system, this point needs to be introduced earlier and more fully (in the Introduction and/or beginning of the Results).

4) The authors mention the wildly oscillatory FRET signal in Figure 6D, but they fail to comment on this further. The authors don't make it clear whether they think reflects a real biological phenomenon, or reflects a technical limitation of measurement of weak FRET signals. Synchronous oscillation of an ensemble of receptors seems implausible, so some comment is necessary.

5) The paper does not mention the other proteins that are recruited to the p75^NTR^ intracellular domain in a neurotrophin-dependent manner (NRIF, TRAF6, etc.). We realize that little can be said at present about how those proteins might influence the death domain interactions described and a full discussion is beyond the scope of the paper, but we would appreciate a discussion of the implication of TRAF6 binding, at least, because of its importance for NF-kB signaling.

6) Data presented in Figure 4B and Figure 4C: What were the concentrations used for each binding curve? It would be convenient for the reader to annotate each SPR binding curve with the corresponding concentrations of the flowed binding partner.

7) Data presented in Figure 2C: Why was this SPR experiment not done in kinetic mode? Please discuss this point.

8) The association 'on-rate' is actually ka and the so-called 'off-rate' should be denoted by kd, with k being a lower case letter and a and d as subscripts. The equilibrium dissociation constant Kd is denoted by a capital K and lower case d in the subscript.

9) Data presented in Figure 3—figure supplement 4 (panel B). What was the concentration series used to obtain the reported kinetic binding data? What binding models did the authors use in SPR data fitting? Please state in each relevant figure legend.

10) Please replace 'sensogram' with 'sensorgram' throughout the manuscript (including the figure legends).

11) It would help the reader tremendously if the authors would come up with a uniform way of annotating the figures presenting binding data to illustrate schematically the geometry of the experiment. For instance, to show which binding partner was immobilized versus the one that was flowed.

12) The authors did not provide (structural) insights as to how the interaction of p75^NTR^-DD with RhoGDI would lower the affinity between RhoGDI and RhoA. Please discuss this point.

13) A general comment on all figures showing structural models: Please label the N- and C-termini of all models shown.

14) The methods pertaining to the preparation of the recombinant proteins used are not described in sufficient detail. Information on construct design and domain delineation needs to be provided as well as chromatographic elution profiles for the protein complexes used in the structural studies.

# Reply: Author response

**DOI:** 10.7554/eLife.11692.023

*Essential revisions:*

*A major concern is that some key mechanistic conclusions in the manuscript are derived from a comparative study of binding affinities of the protein domains, and the mechanistic insights are derived from an SPR-based experimental approach that needs to be revised or at least validated in part by an orthogonal approach. The strategy to immobilize p75^NTR^-DD and RhoA via amine coupling onto CM5 sensor chips for the binding studies via SPR introduces uncertainties about the reliability of the data obtained and the ensuing comparison of affinities. This is because the two proteins have lysine residues at or near the interaction interfaces involved in their complexes as revealed by the structural studies. Given that the coupling to CM5 sensor chips would largely occur via surface exposed lysine residues, any ensuing SPR data generated using such an approach is inherently unreliable as partial steric obstruction of available binding sites would compromise the kinetics and affinities of the interaction under study. The authors appear to possess the necessary tools for readdressing their binding studies, given that they produced the relevant proteins for NMR studies. The Methods section implies, although it does not explicitly state, that recombinant p75^NTR^-DD and RhoA were produced as His-tagged proteins. If that is indeed the case immobilization approaches described in Wear and Wilkinshaw Analytical Biochemistry 371 (2007) 250-252 might provide a convenient alternative for at least repeating some key measurements. Otherwise, isothermal titration calorimetry might provide yet another tool for generating the necessary orthogonal information on affinities, at least for the strongest interactions in the study. Another approach, albeit experimentally more laborious, would be to produce N- or C-terminally biotinylated versions of the proteins of interest and use streptavidin biosensor chips to immobilize the proteins for SPR measurements.*

We have followed the advice of the reviewers and repeated the most critical affinity determinations using the His-tag at the C-terminus of our purified p75^NTR^-DD protein for immobilization to NTA chips. These new measurements are now in the revised version of Table 2 and Figures 4B and 4C. We note that the new equilibrium Kd for interaction of RhoGDI to p75^NTR^-DD is virtually identical to the previous one that was derived from DD protein immobilized by amine coupling. The revised Kd for interaction of RIP2-CARD to p75^NTR^-DD is 1.69-fold lower than the previous one. This indicates that RIP2-CARD binds with approximately 177-fold higher affinity than RhoGDI to the p75_NTR_ DD, result that is very similar to (in fact even larger difference than) the one reported previously using CM5 chips. These results fully support (in fact, strengthen) our mechanistic conclusions concerning competition between these two interactors for p75^NTR^-DD binding.

We note that the Kd for RhoGDI binding to RhoA that was reported in our manuscript (0.14 ± 0.01 µM) is very similar to the one previously published by other groups. For example, Tnimov et al. (2012) reported a Kd of 0.17 µM for this same interaction. Therefore, we are confident that this measurement is correct.

*Minor points (which need to be addressed in the text):*

*1) Why was RhoA-GDP-Mg^2+^ used for the binding studies? What are the biological extrapolations from a complex involving RhoA bound by the product of GTP hydrolysis?*

RhoGDI binds and sequesters RhoA in the inactive, i.e. GDP-loaded, state. Although GTP-loaded RhoA is also able to bind RhoGDI in vitro, the binding affinity of this complex is much weaker and its lifetime much shorter compared to the RhoGDI:RhoA-GDP-Mg^2+^ complex. RhoA-GDP-Mg^2+^ is therefore more suitable for binding studies with RhoGDI protein. In cells, a stable cytosolic pool of GDP-loaded RhoA (inactive form) is largely maintained (or chaperoned) by RhoGDI. In response to specific signals, release from RhoGDI and insertion in the membrane leads to RhoA activation (GTP-loading). Deactivation by GAPs allows GDP-loaded RhoA to be extracted from the membrane by RhoGDI, thus resetting the cycle.

*2) In the first paragraph of the subsection “Solution structure of the complex between the p75^NTR^ DD and RIP2 CARD”, the authors cite a previous study reporting that the interaction between p75^NTR^ and RIP2 is mediated by their DD and CARD domains, yet, in the first paragraph of the Discussion, the authors indicate that the present study is the first heterotypic interaction described in the DD superfamily. These statements are seemingly in contradiction. Certainly the present statement is the first structural characterization of such a complex.*

Point taken. Although the previous study did not used purified proteins, it did already indicate that CARD and DD are sufficient for binding. We have changed this sentence to mean that our present study is the first structural characterization of a heterotypic interaction in the DD superfamily.

*3) p75^NTR^ is somewhat unusual in that ligand binding terminates a constitutively active signaling pathway (RhoA). The first sentence of the Results indicates that the receptor signals through RhoA constitutively, but fails to make it clear that neurotrophin binding terminates this signaling (although they make this point later). To avoid confusing readers who lack familiarity with the p75^NTR^ system, this point needs to be introduced earlier and more fully (in the Introduction and/or beginning of the Results).*

We have followed this advice and introduced this concept earlier in the text of the revised manuscript (Results, first paragraph).

*4) The authors mention the wildly oscillatory FRET signal in Figure 6D, but they fail to comment on this further. The authors don't make it clear whether they think reflects a real biological phenomenon, or reflects a technical limitation of measurement of weak FRET signals. Synchronous oscillation of an ensemble of receptors seems implausible, so some comment is necessary.*

The oscillations observed may indeed reflect a technical limitation of the measurement of weak FRET signals, as suggested by the reviewers. On the other hand, we do think that synchronous oscillation of ensembles of receptors is in fact possible, but this is unlikely what is being detected here, as the average period of these oscillations (2-3 min) would seem too slow to reflect real molecular dynamics which usually are much faster. We have clarified this point in the relevant section of the text (Results, last paragraph).

*5) The paper does not mention the other proteins that are recruited to the p75^NTR^ intracellular domain in a neurotrophin-dependent manner (NRIF, TRAF6, etc.). We realize that little can be said at present about how those proteins might influence the death domain interactions described and a full discussion is beyond the scope of the paper, but we would appreciate a discussion of the implication of TRAF6 binding, at least, because of its importance for NF-kB signaling.*

TRAF6 has been reported to interact with the juxtamembrane region of the intracellular domain of p75^NTR^, but not with its death domain. It is difficult at present to speculate on the possible implications of that interaction for the mechanisms described in the present study, which focus on the death domain. It is possible that such interactions are independent and non-overlapping. RIP2 has been shown to be polyubiquitinated (e.g. Hasegawa et al., 2008 EMBO J. 27:373–83 and Bertrand et al., 2011 PLoS ONE 6, e22356) and we are at present investigating the possible ubiquitination of RIP2 after its association with p75^NTR^. As TRAF6 has ubiquitin ligase activity, there might be a biochemical connection between these two p75^NTR^ effectors. We have included one sentence in the Discussion to highlight this possibility (subsection “A model for the early stages of p75^NTR^ engagement with the RhoA and NF-kB pathways).

*6) Data presented in Figure 4B and Figure 4C: What were the concentrations used for each binding curve? It would be convenient for the reader to annotate each SPR binding curve with the corresponding concentrations of the flowed binding partner.*

The concentrations used have now been indicated on the SPR curves.

*7) Data presented in Figure 2C: Why was this SPR experiment not done in kinetic mode? Please discuss this point.*

Due to weak binding affinity of RhoGDI to p75^NTR^ DD, it was difficult to apply a simple kinetic model to fit the data and get a Kd for binding of the p75^NTR^ DD:RhoGDI complex to RhoA. But it was possible to do this through steady state analysis. As we wanted to make a direct comparison between Figures 2C and 2D, both were done in steady-state mode. We note that the Kd obtained here for the interaction between RhoGDI and RhoA is consistent with previously published results, as indicated above and in the text of our manuscript.

*8) The association 'on-rate' is actually ka and the so-called 'off-rate' should be denoted by kd, with k being a lower case letter and a and d as subscripts. The equilibrium dissociation constant Kd is denoted by a capital K and lower case d in the subscript.*

Thanks. We have changed these accordingly.

*9) Data presented in Figure 3—figure supplement 4 (panel B). What was the concentration series used to obtain the reported kinetic binding data? What binding models did the authors use in SPR data fitting? Please state in each relevant figure legend.*

We have now indicated all the concentrations in the respective SPR curves. One binding site model was used for SPR data fitting. This has now been indicated in the figure legends and Methods section.

*10) Please replace 'sensogram' with 'sensorgram' throughout the manuscript (including the figure legends).*

We have replaced these accordingly.

*11) It would help the reader tremendously if the authors would come up with a uniform way of annotating the figures presenting binding data to illustrate schematically the geometry of the experiment. For instance, to show which binding partner was immobilized versus the one that was flowed.*

We have revised these figures and included the schematics requested.

*12) The authors did not provide (structural) insights as to how the interaction of p75^NTR^-DD with RhoGDI would lower the affinity between RhoGDI and RhoA. Please discuss this point.*

Direct structural insights into this matter would require actual experimental determination of the tripartite DD:RhoGDI:RhoA complex and its comparison to the RhoGDI:RhoA complex. In the absence of this structure, we have examined changes in the RhoGDI structure when in complex with either P75^NTR^ DD or RhoA. As mentioned in the text of our manuscript (and documented in Figure 2—figure supplement 1), this revealed local structural perturbations in RhoGDI upon binding to the DD compared to RhoA. Based on this analysis, we have suggested in the Discussion of our manuscript a potential allosteric mechanism by which small but detectable structural changes in RhoGDI elicited upon binding to the p75^NTR^ DD contribute to the release of RhoA. It is possible that other processes, such as RhoGDI phosphorylation, may also contribute. At this point, and in the absence of additional structural information, we consider that the current discussion of this point is adequate.

*13) A general comment on all figures showing structural models: Please label the N- and C-termini of all models shown.*

We have labeled all the N- and C-termini in the figures.

*14) The methods pertaining to the preparation of the recombinant proteins used are not described in sufficient detail. Information on construct design and domain delineation needs to be provided as well as chromatographic elution profiles for the protein complexes used in the structural studies.*

We have included additional information in the Methods section with details on construct design and delineation of protein domains. Protein complexes were prepared by mixing individual purified proteins at different ratios. We did not use gel filtration to purify the complexes. Chromatographic elution profiles are usually used for complexes of strong binding affinity, it is not suitable to define complexes of weak binding affinity. The binding affinities of DD:RhoGDI and DD homodimer complexes are too weak to display distinct dimer peaks in gel filtration chromatography. Although the affinity of the DD:CARD complex is stronger, the special conditions used for its formation (i.e. pure water) are not suitable for gel filtration which requires at least 150 mM salt in the running buffer to prevent non-specific binding to the matrix. This has now been clarified in the text of the revised manuscript (subsection “Sample preparation”).
